# Influenza Transmission in the Mother-Infant Dyad Leads to Severe Disease, Mammary Gland Infection, and Pathogenesis by Regulating Host Responses

**DOI:** 10.1371/journal.ppat.1005173

**Published:** 2015-10-08

**Authors:** Stéphane G. Paquette, David Banner, Stephen S. H. Huang, Raquel Almansa, Alberto Leon, Luoling Xu, Jessica Bartoszko, David J. Kelvin, Alyson A. Kelvin

**Affiliations:** 1 Division of Experimental Therapeutics, Toronto General Hospital Research Institute, University Health Network, Toronto, Ontario, Canada; 2 Institute of Medical Science, Faculty of Medicine, University of Toronto, Toronto, Ontario, Canada; 3 Department of Immunology, Faculty of Medicine, University of Toronto, Toronto, Ontario, Canada; 4 Infection and Immunity Medical Investigation Unit, Hospital Clínico Universitario—Instituto de Estudios de Ciencias de la Salud de Castilla y Leόn, Valladolid, Spain; 5 Sezione di Microbiologia Sperimentale e Clinica, Dipartimento di Scienze Biomediche, Universita' degli Studi di Sassari, Sassari, Italy; 6 International Institute of Infection and Immunity, Shantou University Medical College, Shantou, Guangdong, China; 7 Guangdong Provincial Key Laboratory of Infectious Diseases and Molecular Immunopathology, Guangdong, China; 8 Immune Diagnostics & Research, Toronto, Ontario, Canada; St. Jude Children's Hospital, UNITED STATES

## Abstract

Seasonal influenza viruses are typically restricted to the human upper respiratory tract whereas influenza viruses with greater pathogenic potential often also target extra-pulmonary organs. Infants, pregnant women, and breastfeeding mothers are highly susceptible to severe respiratory disease following influenza virus infection but the mechanisms of disease severity in the mother-infant dyad are poorly understood. Here we investigated 2009 H1N1 influenza virus infection and transmission in breastfeeding mothers and infants utilizing our developed infant-mother ferret influenza model. Infants acquired severe disease and mortality following infection. Transmission of the virus from infants to mother ferrets led to infection in the lungs and mother mortality. Live virus was also found in mammary gland tissue and expressed milk of the mothers which eventually led to milk cessation. Histopathology showed destruction of acini glandular architecture with the absence of milk. The virus was localized in mammary epithelial cells of positive glands. To understand the molecular mechanisms of mammary gland infection, we performed global transcript analysis which showed downregulation of milk production genes such as Prolactin and increased breast involution pathways indicated by a STAT5 to STAT3 signaling shift. Genes associated with cancer development were also significantly increased including JUN, FOS and M2 macrophage markers. Immune responses within the mammary gland were characterized by decreased lymphocyte-associated genes CD3e, IL2Ra, CD4 with IL1β upregulation. Direct inoculation of H1N1 into the mammary gland led to infant respiratory infection and infant mortality suggesting the influenza virus was able to replicate in mammary tissue and transmission is possible through breastfeeding. In vitro infection studies with human breast cells showed susceptibility to H1N1 virus infection. Together, we have shown that the host-pathogen interactions of influenza virus infection in the mother-infant dyad initiate immunological and oncogenic signaling cascades within the mammary gland. These findings suggest the mammary gland may have a greater role in infection and immunity than previously thought.

## Author Summary

Influenza is known as a respiratory infectious disease. Breastfeeding allows for frequent microbial exchange between infant and mother. Although infants, pregnant women and breastfeeding mothers are more susceptible to severe respiratory disease following influenza virus infection, the mechanisms of disease severity in infants and mothers is poorly understood. We were interested in understanding the immune responses, pathogenicity, and transmission dynamics in the infant-mother system. With this aim we developed an infant-mother ferret influenza model. Influenza infection in infants led to virus transmission to mothers causing severe disease and mortality. Unexpectedly, influenza-infected infant ferrets transmitted the virus to mother mammary glands leading to live influenza virus in expressed breast milk. Gene regulation analysis in the mammary gland showed reduction of milk production genes such as Prolactin and increased genes involved with breast shutdown. Genes associated with cancer development were significantly increased including *JUN*, *FOS* and *BRCA2*. We found human breast cells were able to be infected with the influenza virus suggesting the importance of our results to human health. Our data suggests influenza transmission can occur through the mammary glands initiating immunological and pathological events contributing to influenza disease severity impacting infant and maternal health.

## Introduction

The influenza A RNA virus (Orthomyxoviridae) is a significant threat to human health and the global economy by causing morbidity and mortality through frequent epidemics and sporadic pandemics [1]. Seasonal influenza viruses such as seasonal H1N1 and H3N2 infect the cells lining the upper respiratory tract. These viruses typically cause mild clinical symptoms including rhinorrhea, fever, and myalgia [2]. Unlike seasonal influenza, highly pathogenic viruses such as avian H5N1 are able to infect organs outside the upper respiratory tract such as the lower lungs, intestines, and brain, causing a more severe illness [3]. The localization of influenza virus infection is largely influenced by the distribution of the virus cellular receptors where the α2,6-linked sialic acid receptor is predominantly expressed in the upper respiratory tract and α2,3-linked sialic acid receptors in the lower [4]. In 2009, a novel variant pandemic H1N1 influenza A (2009 H1N1) virus emerged from Mexico that targets both the upper and lower respiratory system [4]. During the 2009 H1N1 pandemic, infants, nursing-mothers, and pregnant women were considered to be at high risk of developing severe disease [5–7] and viral or bacterial pneumonia [8]. Pediatric and pregnant/postpartum mother mortalities were sharply increased in the 2009–2010 influenza season compared to previous seasons [9,10]. Importantly, the underlying mechanisms leading to severe disease in mothers and infants have not been elucidated, suggesting the need for an animal model.

The mother-infant dyad represents a unique relationship where the infant and mother are often considered to function as a single unit. Through breastfeeding, mothers provide nutrients, beneficial bacteria, and immune protection to the infant by transfer of cells, antibodies, proteins, and sugars through breast milk [11,12]. The consumption of breast milk has a central role establishing infant health and disease susceptibility by influencing the infant gastrointestinal and immune systems [13,14]. The maternal breast is a highly structured organ and an area of frequent traffic between infant and mother. Fluid exchange also occurs from infant to mother during breastfeeding as liquid can infiltrate the mammary gland through retrograde flux of the nipple [15]. It is currently thought that maternal plasma cells producing antibodies for breast milk delivery are a consequence of cellular migration from the maternal gut to the breast without a host-pathogen interaction occurring within the mammary gland [16]. Although most infants worldwide are breastfed, little is known regarding pathogen transmission between nursing-mothers and infants, breast susceptibility to pathogens, or the local immune response within the breast due to lack of a translatable research model [5].

Breast disease is a significant problem worldwide. The postpartum and lactation phase in breast metamorphosis has been associated with increased breast cancer incidence. While breastfeeding has been shown to protect against long-term breast cancer development, postpregnancy breast cancer is hypothesized to be a consequence of breast remodeling and involution associated with lactation [17,18]. Infectious breast tumor etiologies have been hypothesized as nucleic acids from viruses, such as human papilloma virus (HPV) and Epstein-Barr virus (EBV), have been recovered from within breast tumors [19–22]. These viruses express oncoproteins that inactivate the host’s basic cellular activity by blocking tumor repressor proteins or promote cellular transformation [23]. Furthermore, these viruses may have a significant latency period allowing long periods of viral presence without clearance.

Although infants and mothers are highly susceptible to the influenza virus [24–27], little is known regarding mother-infant transmission and susceptibility of breast tissue. We developed a ferret model of influenza transmission in the mother-infant dyad (infant-to-mother ferret influenza transmission model) with the aim of investigating virus transmission and immune consequences during influenza infection in this highly susceptible population. Ferrets are considered to be the most appropriate animal model for human influenza pathogenesis, vaccine optimization, and antiviral development [28,29]. Ferrets are also used to investigate transmissibility of the influenza virus due to the similarity of the ferret and human respiratory biology [29–31]. Previously, we developed both “young” and “aged” ferret models of influenza pathogenesis due to shared characteristics of development and aging between ferrets and humans [32,33]. Building on these studies, we developed a novel infant-mother ferret influenza model utilizing nursing-mother ferrets and their 4-week-old feeding-infants (infant-mother ferret influenza transmission model) to investigate the host-pathogen interactions of influenza virus infection in the mother-infant dyad. Our investigation showed established virus infection within the mammary gland and viral shedding in breast milk suggesting a novel mechanism of influenza virus transmission.

## Results

### Severe morbidity, mortality, and respiratory disease following influenza transmission from infant to mother

**Fig 1:**
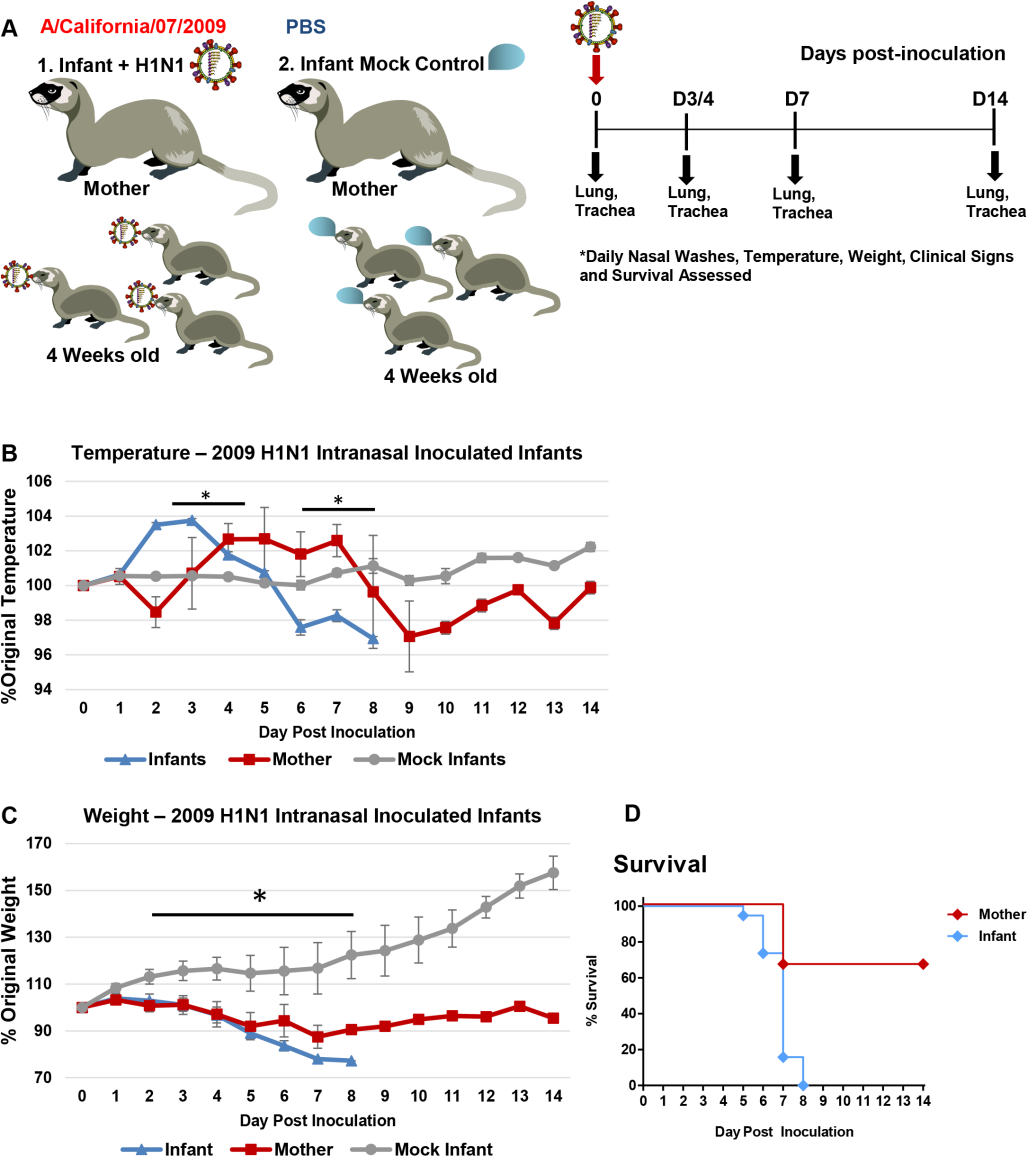
Intranasal 2009 H1N1 infection in infant ferrets leads to severe disease and mortality in both mother and infant ferrets. Schematic of experimental design for mother-infant dyad inoculations (**A**). Infants were intranasally inoculated with the A/Cal strain of 2009 H1N1 influenza (10^5^ EID_50_) and housed with their nursing-mothers. Temperature (**B**) and weight (**C**) were recorded for 14 days post-infant-inoculation. Control mock inoculated infants (grey lines) were used to assess natural fluctuations in growing infants. Survival of mothers and infants was determined over 14 days (**D**). Results show the mean or are representative of 3 independent litter inoculations/infections (3 mother ferrets and 19 infant ferrets) and 3 litter mock inoculations (3 mother ferrets and 11 infant ferrets). * indicates a p-value less than 0.05 determined by ANOVA comparing 2009 H1N1 inoculated/infected to mock controls. Error bars indicate +/- SE.

To investigate influenza infection in the mother-infant dyad, we intranasally inoculated 4-week-old infant ferrets with the 2009 H1N1 virus, A/California/07/2009 (Cal/07), at 10^5^ EID_50_. Infants were housed with nursing-mothers before and after inoculation. Nasal washes (NW), temperature, weight, clinical assessment, and survival were collected daily as shown in the design schematic (**Fig 1A**). Necropsies were performed at Day 3, 4, 7, and 14 post-inoculation or if an animal was removed from the study (reached weight cut-off or had succumbed to illness). Inoculated infant ferrets developed illness leading to mortality starting with a high fever at 104% of baseline temperature (p<0.05) Day 2 post-infant-inoculation. Infants then developed hypothermia (Day 6) (**Fig 1B**). All infants reached weight cut-off (lost 20% of original weight; p<0.05) or died by Day 8 post-infant-inoculation (**Fig 1C and 1D**). Four days post-infant-inoculation, nursing-mother ferrets also developed influenza-like symptoms including increased body temperature (103% of baseline). Mother weights significantly decreased by 13% Day 4 post-infant-inoculation (**Fig 1C**). Weight did not recover by study end and one mother reached cut-off (**Fig 1D**). The weight and temperature of the mothers of mock inoculated control infants are presented in **S1 Table** which showed mothers did not develop symptoms post-mock-inoculation of infant ferrets.

**Fig 2:**
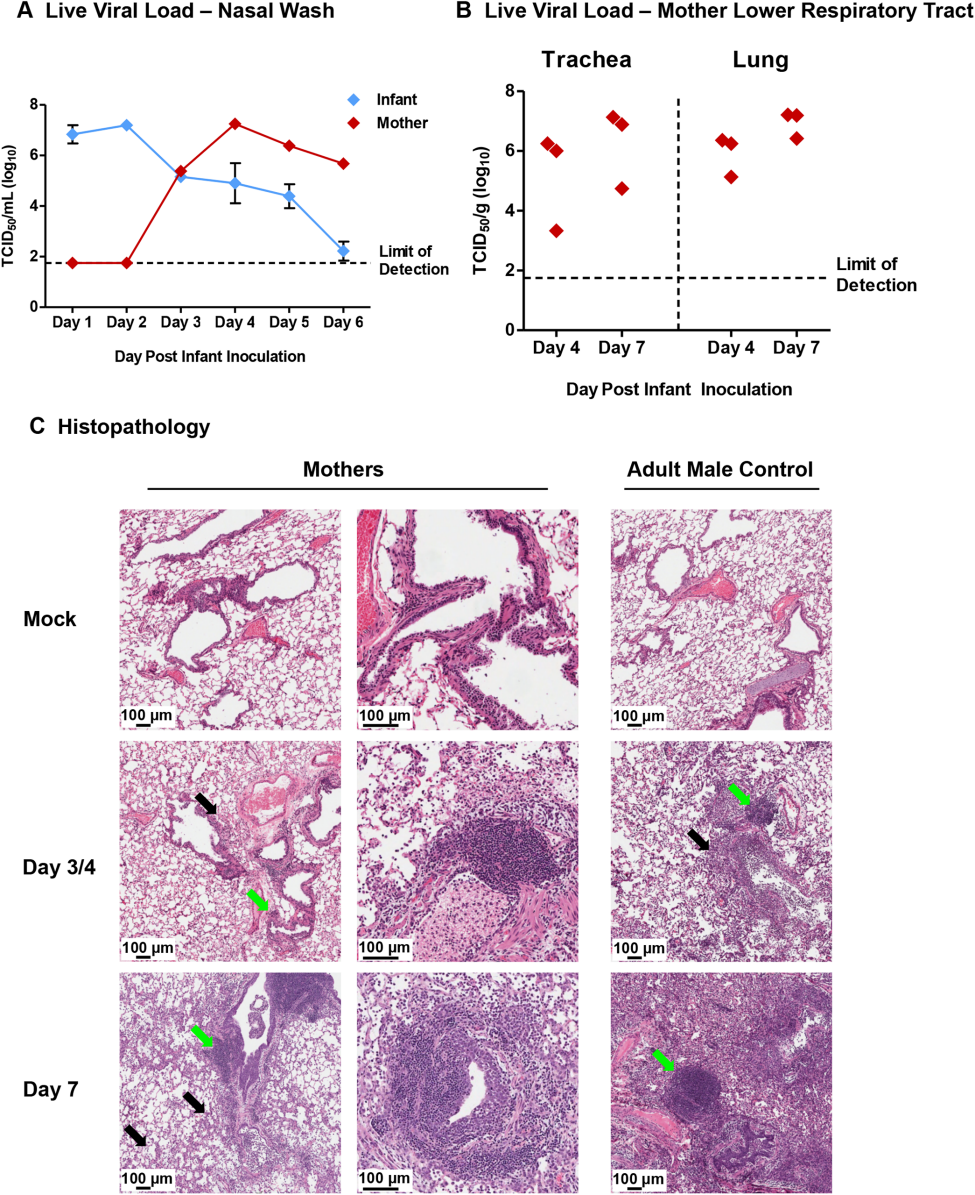
Transmission of H1N1 influenza from infants to mother ferrets causes upper and lower respiratory tract infection with significant pathology. Inoculated infants (A/Cal H1N1 influenza (10^5^ EID_50_)) were housed with their nursing-mothers. Nasal washes were collected daily from infants and mothers. Live viral loads were determined by MDCK titration assay in nasal wash (NW) (**A**) and mother trachea and mother lungs at specific time points (**B**). Lungs were harvested on Day 3/4 and 7 post-infant-inoculation for hematoxylin & eosin (H&E) histopathological assessment from nursing-mothers of inoculated infants and directly inoculated adult ferrets as control (**C**). Green arrows denote dense cell accumulation; black arrows denote diffuse immune cell infiltration. Black arrows not included on Day 7 adult tissue due to widespread infiltration. High resolution scans were performed using an Aperio ScanScope XT, Leica Biosystems, Nußloch, Germany. The left and central columns of mother images represent a low and high magnification of each lung scan. The scale bars indicate the relative 100 μm. Results of **A** are representative of 3 independent litter inoculations where nasal washes were collected from 3 mothers and 9 infants (3 per litter) daily. Results of **B** and **C** are from 6 independent litter inoculations of serial tissue collections (3 collected Day 3/4 and 3 collected Day 7). Error bars indicate +/- SD. Mock: mother ferrets nursing mock-inoculated 4-week-old infant ferrets. Results show the mean or are representative of independent litter inoculations/infections.

As the mothers of inoculated infants began to display clinical signs of influenza including temperature increases, weight loss, and mortality, we went on to analyze the upper and lower respiratory tracts of the infants and their mothers to determine virus transmission and pathogenesis. Viral burden in infant NW was first detected Day 1 post-infant-inoculation (**Fig 2A**). The NW of nursing-mothers were positive for influenza virus Day 3 post-infant-inoculation. Viral titers remained high in mother NW Day 6–7 (**Fig 2A**) at more than 6 TCID_50_/ml (Log_10_). Residual virus persisted in the mothers’ NW by Day 10 (**S1A Fig**). Virus transmission between adult ferrets pair-housed (**S1C Fig**) were used as a comparison of transmission dynamics between infants and mother ferrets. For this control, one adult was intranasally inoculated under similar circumstances as the infant-mother inoculations and NW were collected for viral load assessment. Naïve cage mates of inoculated adult ferrets began shedding virus in NW Day 3 post-inoculation. These results showed that the influenza virus was able to be transmitted from direct intranasally inoculated infant ferrets to the upper respiratory tracts of their nursing-mothers on a similar time scale as that of adult ferrets.

We next determined if transmission of the virus from infants to mother led to lower respiratory tract infection. Examination of viral titers in the lower respiratory tract of the mothers found virus levels above 6 TCID_50_/ml (Log_10_) in both the trachea and lungs post-infant-inoculation in 2 out of 3 mothers investigated (Day 4 and 7 post-infant-inoculation) (**Fig 2B**). H&E (hematoxylin & eosin) staining of mother lungs revealed delayed, airway-localized inflammation (**Fig 2C**). Day 3/4 post-infant-inoculation, mother lungs showed areas of minimal infiltrating leukocytes (black arrows: diffuse areas; green arrows: dense infiltration) (**Fig 2C**). Increased leukocyte infiltration was observed Day 7 post-infant-inoculation where sites of inflammation were dense in mononuclear cells with lymphocyte-like morphology. Infected mother lungs were compared to the lungs of adult male ferrets directly inoculated with Cal/07 through the intranasal route (**Fig 2C**, right column). The lungs collected from mothers of mock inoculated infants were similar in architecture to that of mock inoculated adult male ferrets. The adult lungs collected Day 3/4 post-direct-inoculation had markedly more leukocyte infiltrates compared to the mother lungs at Day 3/4 post-infant-inoculation (**Fig 2C**). Taken together with the above data, these findings indicated that 2009 H1N1 transmission from infant to mother led to severe respiratory disease with upper and lower respiratory tract infection and accompanying lung pathology but on a delayed time course compared to direct adult infections.

### Influenza virus transmission also occurs mother to infant

**Fig 3:**
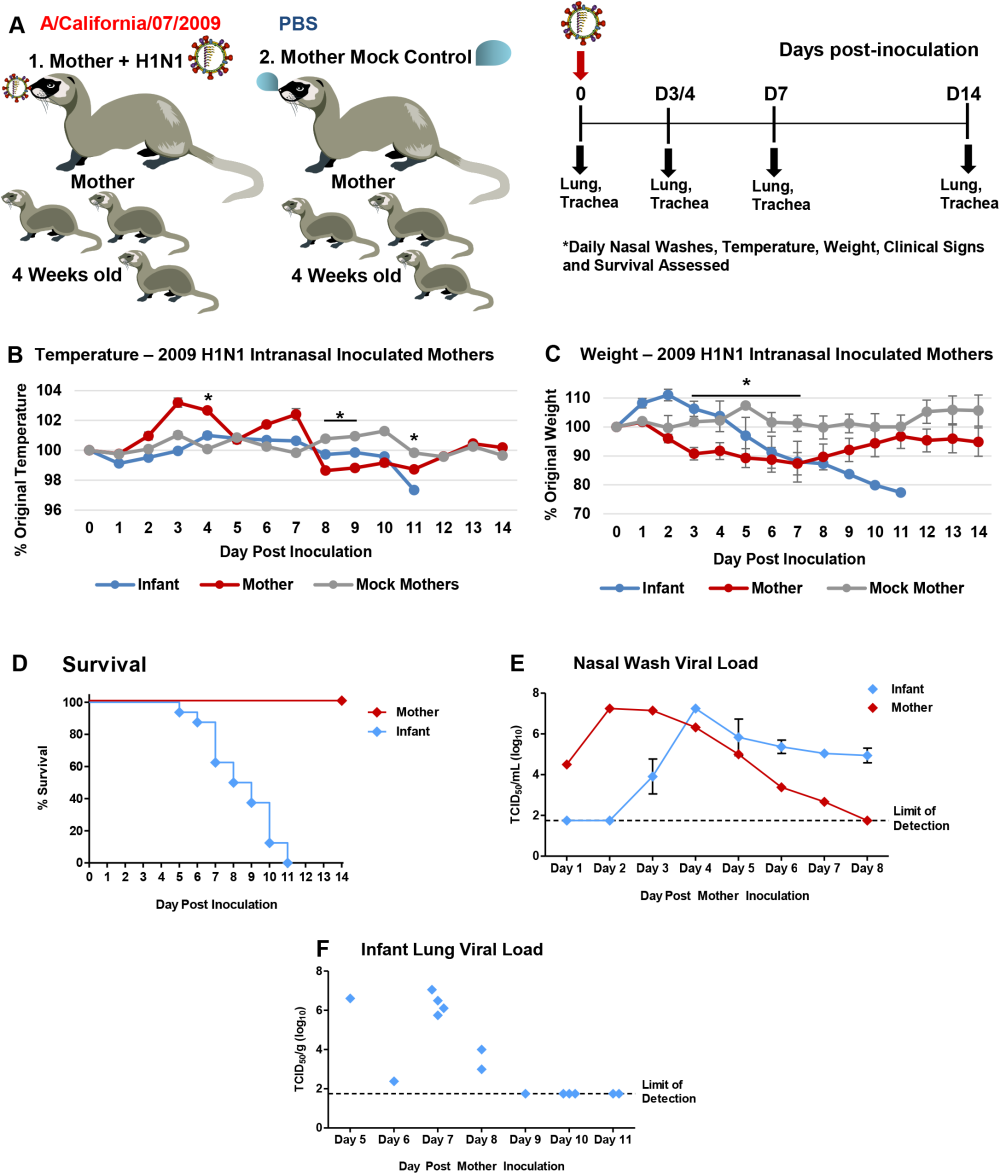
Intranasal 2009 H1N1 infection in mother ferrets leads to severe disease and virus transmission to breastfeeding infants. Schematic of nursing-mothers intranasally inoculated with the Cal/07 strain of 2009 H1N1 influenza (10^5^ EID_50_) and housed with their feeding infants (**A**). Temperature (**B**) and weights (**C**) were recorded daily for mothers and infants post-inoculation. Grey lines indicate mock inoculated mothers. Survival of mothers and infants post-mother-inoculation (**D**). Live viral loads were quantified by MDCK titration of nasal wash (NW) (**E**) and infant lung (**F**) homogenates collected throughout the infection course (infants that had reached cut-off or succumbed to illness). * indicates a p-value less than 0.05 determined by ANOVA comparing H1N1 inoculated to mock controls. Error bars indicate +/- SD. Data was collected from three independent litter inoculations/infections (3 inoculated/infected mothers, 16 infants, and 3 mock inoculated/infected mothers) and results show the mean or are a representative of the inoculations/infections.

**Fig 4:**
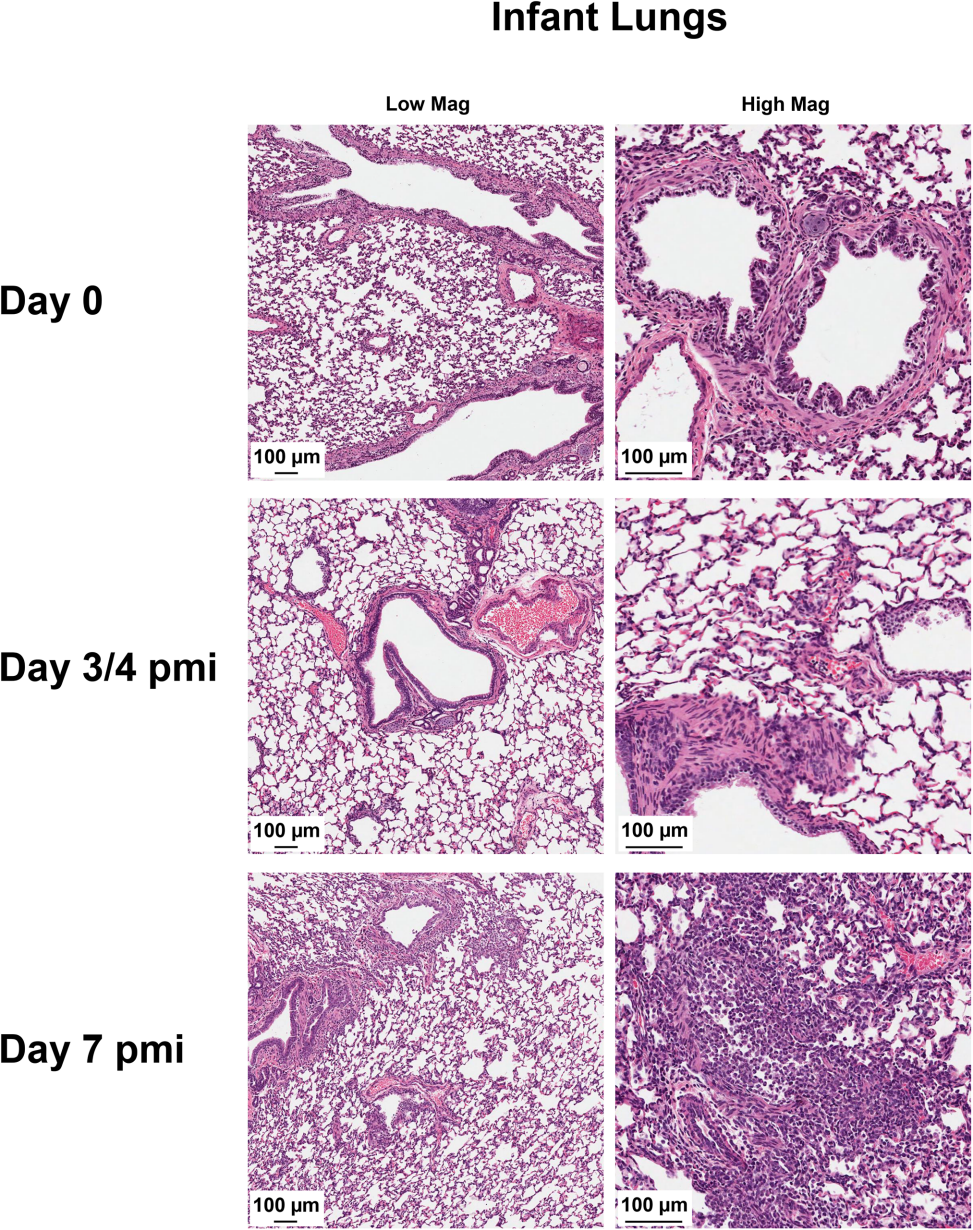
2009 H1N1 transmission from mothers to infants results in severe lower respiratory tract pathology. Harvested lungs from control infants and infants of inoculated nursing-mothers were processed for histopathological assessment. Tissue morphology was assessed by hematoxylin & eosin staining. Data was collected from three independent litter inoculations/infections (3 inoculated/infected mothers, 16 infants, and 3 mock inoculated/infected mothers) and results are a representative of the inoculations/infections. pmi = Post-Mother-Inoculation

To determine if influenza infected nursing-mother ferrets could transmit the virus to their feeding-infants, we reversed the inoculation/infection design as shown in the schematic (**Fig 3A**). We intranasally inoculated nursing-mothers with Cal/07 at 10^5^ EID_50_ and observed clinical symptoms as well as determined respiratory viral load and pathogenesis. Following inoculation, nursing-mother ferrets developed a biphasic fever which spiked at 104% (baseline temperature) (**Fig 3B**) Day 3 post-mother-inoculation. Weight loss was observed in the inoculated mothers which reached nadir on Day 7 post-mother-inoculation at 89% of original weight (p<0.05) (**Fig 3C**). Although mother mortality did not occur, the infants of inoculated mothers lost weight, had increased temperature, and all died or reached cut-off by Day 11 post-mother-inoculation (**Fig 3D**). The weight of infants of inoculated nursing-mothers began to decline on Day 3 (**Fig 3C**). Although infants did not have a significant increase in temperature they did become hypothermic as they reached mortality (**Fig 3B**). Virus was detected in mother NW Day 1 post-mother-inoculation (peak Day 2 at 6 TCID_50_/ml (Log_10_)) and infant viral shedding began Day 3 (**Fig 3E**). Live influenza virus was detected in the lungs of infants reaching weight cut-off or succumbing to illness between Day 5 and Day 8 post-mother-inoculation (3–8 TCID_50_ (Log_10_)) (**Fig 3F**). Significant pathology was also detected in lungs of infants feeding from 2009 H1N1 inoculated nursing-mothers Day 7 post-mother-inoculation. The lungs were characterized by areas of dense leukocyte infiltration and alveoli destruction (**Fig 4**). In summary, these data indicate that influenza transmission was also possible from mother to feeding-infants causing upper and lower respiratory tract infection and respiratory tract disease.

### Infants transmit the influenza virus to mother mammary glands

**Fig 5:**
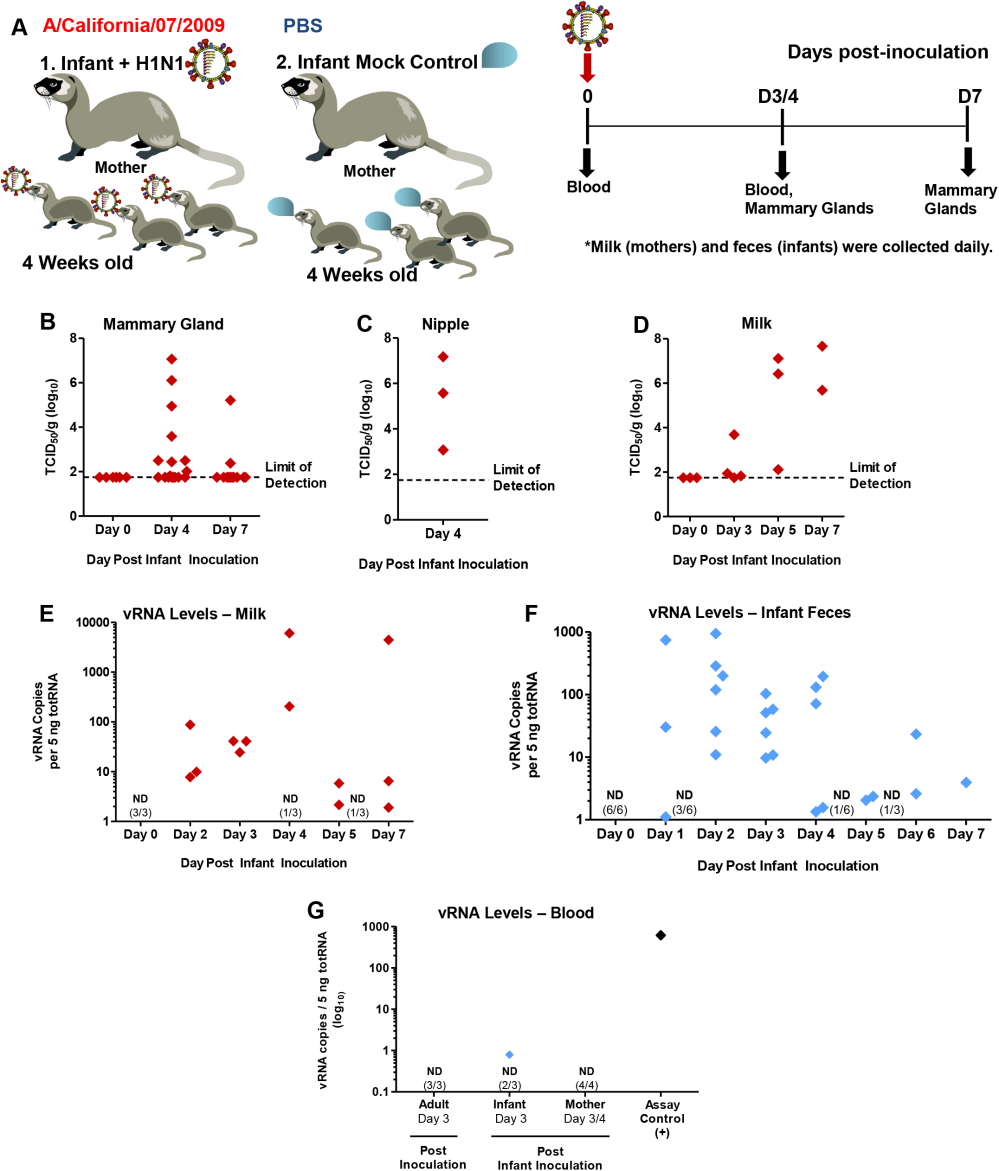
Virus replication and pathology in mammary glands of mothers nursing 2009 H1N1-infected infants. Infant ferrets were intranasally inoculated with A/Cal and housed with mother ferrets for a 7 Day time course where mammary glands, milk, blood, and feces were collected as shown in the schematic (**A**). Live virus was quantified by MDCK titration of homogenized mammary tissue (**B**), nipples (**C**), and expressed milk (**D**) from nursing-mothers of intranasal inoculated infant ferrets. qRT-PCR was performed on expressed milk for 2009 H1N1 viral RNA (vRNA) (**E**). Viral presence was also determined by qRT-PCR in infant feces (post-direct-inoculation) (**F**) and infant, mother, and adult ferret blood (3/4 days post-inoculation) (**G**). ND = Not Detected. Samples were collected and analyzed from at least 3 independent litter inoculated/infected infants and 3 mothers per time point. Results show the mean or are representative of 3 litters per time point. Mother ferrets have variable numbers of active mammary glands per pregnancy/postpartum.

Mammary glands are a significant point of contact and possible pathogenic transmission within the mother-infant dyad [34]. To investigate the susceptibility of mammary glands to influenza infection and the mammary as a point of transmission, we determined virus presence within mammary tissue and virus shed in expressed milk as shown in the experimental design graphic (**Fig 5A**). All mammary glands were collected from mothers of directly influenza inoculated infants on Day 4 and Day 7 post-infant-inoculation (3 per time point). Live viral load was quantified from each mammary tissue. MDCK titration assay revealed live virus in 7 of the glands (**Fig 5B**) where some viral loads reached 6 TCID_50_/g (Log_10_) or higher. All mothers assessed had at least one gland positive for live influenza virus. Nipples of positive mammary glands also had significant amounts of live influenza virus on Day 4 post-infant-inoculation (3–7 TCID_50_/g (Log_10_)) (**Fig 5C**).

To determine if influenza virus positive mammary glands were able to shed virus in the milk expressed from the infected mammary gland, we measured both live virus and vRNA in expressed milk from mothers of infected infants. Live virus assessment in milk collected between Day 3 and Day 7 post-infant-inoculation revealed virus presence where some samples contained high virus levels between 6–7 TCID_50_ (Log_10_) (**Fig 5D**). vRNA analysis from milk collected on separate occasions showed vRNA was present in some milk samples as high as ~10,000 copies/5 ng totRNA. Most samples collected had between 10 and 1000 copies (**Fig 5E**). vRNA and live virus were not detected in milk collected at baseline (**Fig 5D and 5E**). High levels as well as moderate and low levels of vRNA were detected in infant ferret feces (**Fig 5F**). Negligible or no amounts of vRNA were found in the peripheral blood of intranasally inoculated infants or their mothers to account for viremia contributing to severe disease or virus shedding from milk (**Fig 5G**). Control directly inoculated adults were also negative for blood vRNA. This data suggested that mammary glands are able to be harbor live influenza virus and viral shedding can occur through milk expression.

**Fig 6:**
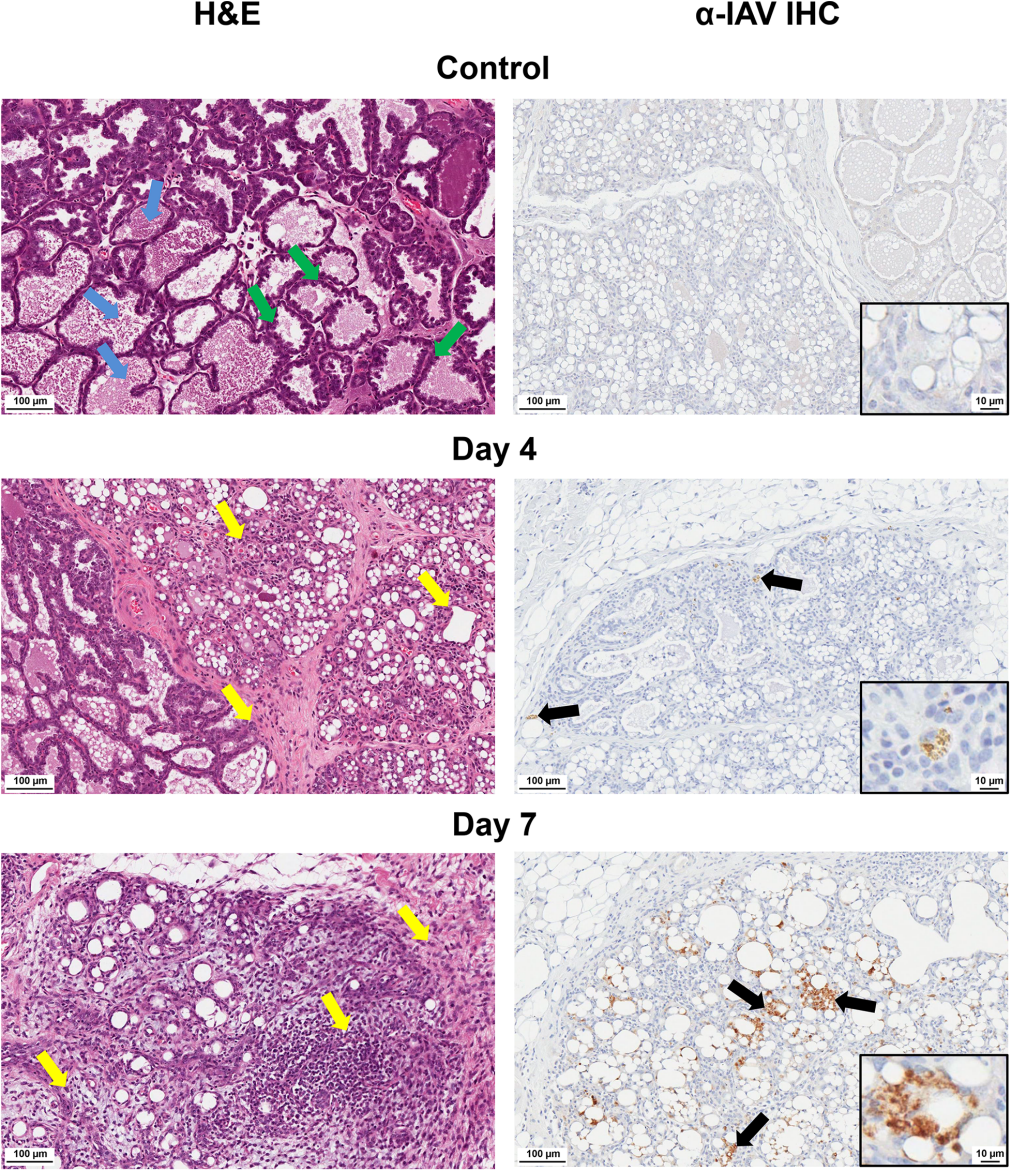
Destruction of acini and accumulation of viral protein in mammary glands of mothers of infected infants. Pathological destruction was determined by H&E (left) and IHC for IAV (right) analysis of paraffin-embedded mammary sections from nursing-mothers of infected infants. Black arrows denote areas of virus expression determined by anti-IAV staining. Yellow arrows denote areas of leukocyte infiltration. Blue arrows show milk protein accumulation. Green arrows indicate active epithelial cell layers. Control = mammary glands from nursing-mothers of mock inoculated infants. Samples were collected and analyzed from at least 3 independent litter inoculated/infected infants and 3 mothers per time point. Results show are representative of 3 litters per time point. Mother ferrets have variable numbers of active mammary glands per pregnancy/postpartum.

We next visualized the virus in 2009 H1N1 influenza positive mammary glands (H1N1+ MG) to determine virus localization and gland pathology following infection. Histopathology and virus localization in H1N1+ MG revealed destruction of mammary architecture (**Fig 6**). The acini of control glands had either thick epithelial cell layers indicative of active milk production (green arrows) or acini filled with pink proteinaceous fluid (milk) (blue arrows) (**Fig 6**). Virus staining was not detected in Control mammaries (top right panel). At Day 4 post-infant-inoculation, the glands of nursing-mothers had retained tissue architecture although leukocyte infiltration (yellow arrows) was identified. Foci of virus staining (light brown in color) was also observed (Day 4 post-infant-inoculation). By Day 7, marked increases in leukocyte infiltration (yellow arrows) in and surrounding the lobules was noted in some mammary glands (**Fig 6**) where the lobules of these glands had lost typical mammary acini architecture (bottom left panel). Increased viral staining was seen by Day 7 post-infant-inoculation with loss of significant tissue structure (bottom right panel). Viral staining was more pronounced in epithelial cells (black arrows) (semi-quantitative analysis). These data provide evidence that the influenza virus is capable of infecting cells of the mammary gland.

### Downregulation of milk production gene signaling and increased oncogene pathways in infected mammary glands

**Fig 7:**
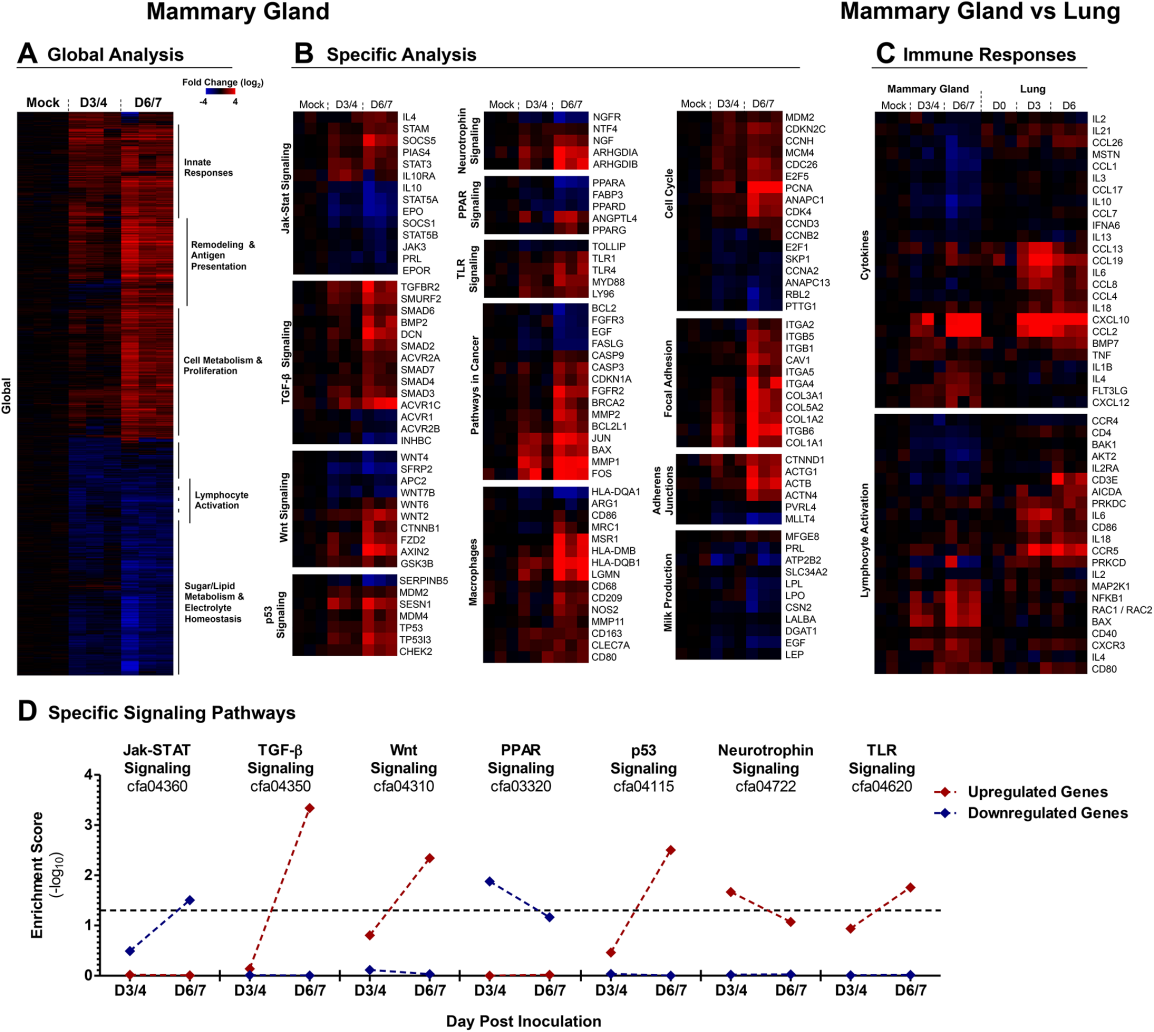
2009 H1N1 virus positive mammary glands have distinct genetic signatures linked to the regulation of milk production, cancer and immune responses. A clustergram of the global expression analysis of H1N1+ MGs is shown and the most prominent functional groups are indicated for each cluster (**A**). Clustergrams of H1N1+ MG gene expression for specific signaling pathways and gene networks were produced (described in methods) (**B**). Immune Responses were analyzed by comparing the genes expression profiles of 2009 H1N1 infected Adult Ferret Lungs against the H1N1+ MGs (**C**). Clustergrams for immune response analysis were generated with genes exhibiting statistically significant differential regulation in either ferret mammary glands or lungs. Gene enrichment scores for KEGG-defined signaling cascades among significantly upregulated (red) and downregulated (red) gene subsets at Days 3/4 and Days 6/7 are shown (**D**). Values above threshold (α = 0.05) indicate statistically significant enrichment among upregulated or downregulated gene subsets at a given time-point. Samples were collected and analyzed from 3 independent litter experiments of inoculated/infected infants and 3 mothers per time point.

To uncover the molecular mechanisms of influenza infection within the mammary glands we performed gene expression analysis on RNA isolated from H1N1+ MGs. Global gene expression analysis by hierarchical clustering using Pearson correlation revealed increased expression of innate immune and cell remodeling genes, as well as downregulation of metabolism and lymphocyte activation genes (**Fig 7A**).

Kyoto Encyclopedia of Genes and Genomes (KEGG) Pathway analysis showed pronounced alterations in signaling gene expression in H1N1+ MGs (**Fig 7B;** Ingenuity Pathway Analysis **S2 Fig**, Gene Enrichment Scores **S2 Table**). *Jak-STAT* (Janus Kinase-Signal Transducers and Activators of Transcription) signaling was prominently affected, highlighted by increased *STAT3* and involution signaling (*STAT3*, *PIAS4*, and *SOCS5*). Corresponding decreases were seen in *STAT5A/B* milk production and STAT associated gene networks *PRL* (Prolactin), *STAT5A/B*, *SOCS1* (Suppressors of Cytokine Signaling 1), *JAK3*, *EPO* (Erythropoietin), and *EPOR* (Erythropoietin Receptor)) [35–38]. As well as *PRL*, other genes involved in milk production [39], *LPO* (Lactoperoxidase), *LPL* (Lipoprotein lipase), *ATP2B2* (ATPase for Ca2+ transport during milk production), *SLC34A2* (protein component of milk), *LALBA* (alpha-lactalbumin), and *CSN2* (β-casein), were also significantly downregulated (**Fig 7B**).

Cancer Related and Cell Cycle genes including *Wnt*, *p53*, and *TGFβ* signaling pathways, as well as cell attachment (Focal Adhesions and Adherens Junctions) gene networks, were regulated in H1N1+ glands. Significant expression of M2 macrophage genes were found including *CD163*, *CLEC7A* (C-type lectin domain family 7, member A), *MSR1*, *CD209*, *LGMN* (Legumain), and *MRC1* (Mannose receptor C type 1) transcripts (**Fig 7B**). *MMP1*, *MMP2*, *MMP8*, *MMP11*, and *MMP14* matrix metalloproteases were also increased along with collagen genes *COL5A2*, *COL3A1*, *COL1A1*, *COl1A2*, *COL4A1*, *COL6A3*, and *COL4A6* (**Fig 7B** and **S2 Fig**). Upregulation of Integrins (*ITGA4*, *ITGB5*, *ITGB1*, *ITGB6*) and other genes involved in Focal Adhesions and Adherens Junctions (*CAV1 (*Calveolin 1*)*, *RAP1A*, *PAK1* (p21 Activated Kinase), *ROCK1* (Rho Kinase), *VAV1* (guanine nucleotide exchange factor), *WASL* (Wiskott-Aldrich Syndrome-Like), *SPPL1*) was also noted (**Fig 7B** and **S2 Fig**). Gene networks involved in Cell Cycle and Cell Proliferation/Stress were also prominent. These included MAP Kinase associated protein genes (*MAP2K3*, *MAP2K6*, *MAPK8*, *MAPK13*, *MAPK1*, *MAP2K1*, *MAP2K4*, *JUN*, and *FOS*) and Cyclin regulation genes (*CDC16*, *CD4*, *CDKN2D*, *CDKN2D*, *CDKN1A*, *CDC26*, *CDK7*, *CDC45*) (**S2 Fig**). Many of these genes have known roles in cancer gene networks such as the proto-oncogenes *JUN* and *FOS*, *RAP1A* (RAS-related protein 1A), *ITGB5*, as well as *BRCA2* (Breast cancer 2, early onset) and *PCNA* (Proliferating cell nuclear antigen) (**Fig 7B** and **S2 Fig**). The *P53* gene network included both pro- and anti-apoptotic gene upregulation (*Bax*, *CASP9* (Caspase 9), *CASP3* (Caspase 3), *PTEN* (Phosphatase and Tensin homolog), *CDK4*, and *MDM4* (Mouse Double Minute 4)).

Functional annotation analysis by KEGG classification of significantly upregulated or downregulated genes similarly suggested pronounced transcription-level changes in cellular proliferation, remodeling, and metabolic pathways in H1N1+ MG. Genes associated with cell growth, morphology, and catabolism were significantly enriched among upregulated genes while genes implicated in lipid and protein metabolism were dominant among downregulated gene subsets (**S2 Table**). Seven signaling cascade gene classifications exhibited statistically significant enrichment among upregulated or downregulated genes for at least one time-point (**Fig 7D**). Among upregulated genes, cell growth (*Wnt*, *TGF-β*) and stress response (*p53*) signaling pathway genes were significantly enriched by Days 6/7 with a trend of increasing enrichment over time. A similar profile was observed for TLR signaling-associated genes. Conversely, genes implicated in *Jak-STAT* and *PPAR* signaling were significantly enriched among downregulated genes, with potential implications for milk production (**Fig 7D**). Microarray gene expression was validated by qRT-PCR for 9 ferret specific primer sets including *STAT3*, *STAT5*, *FOS*, *CSN2*, and *CXCL10* (**S3 Fig**, Ferret Primer Sequences **S3 Table**).

**Fig 8:**
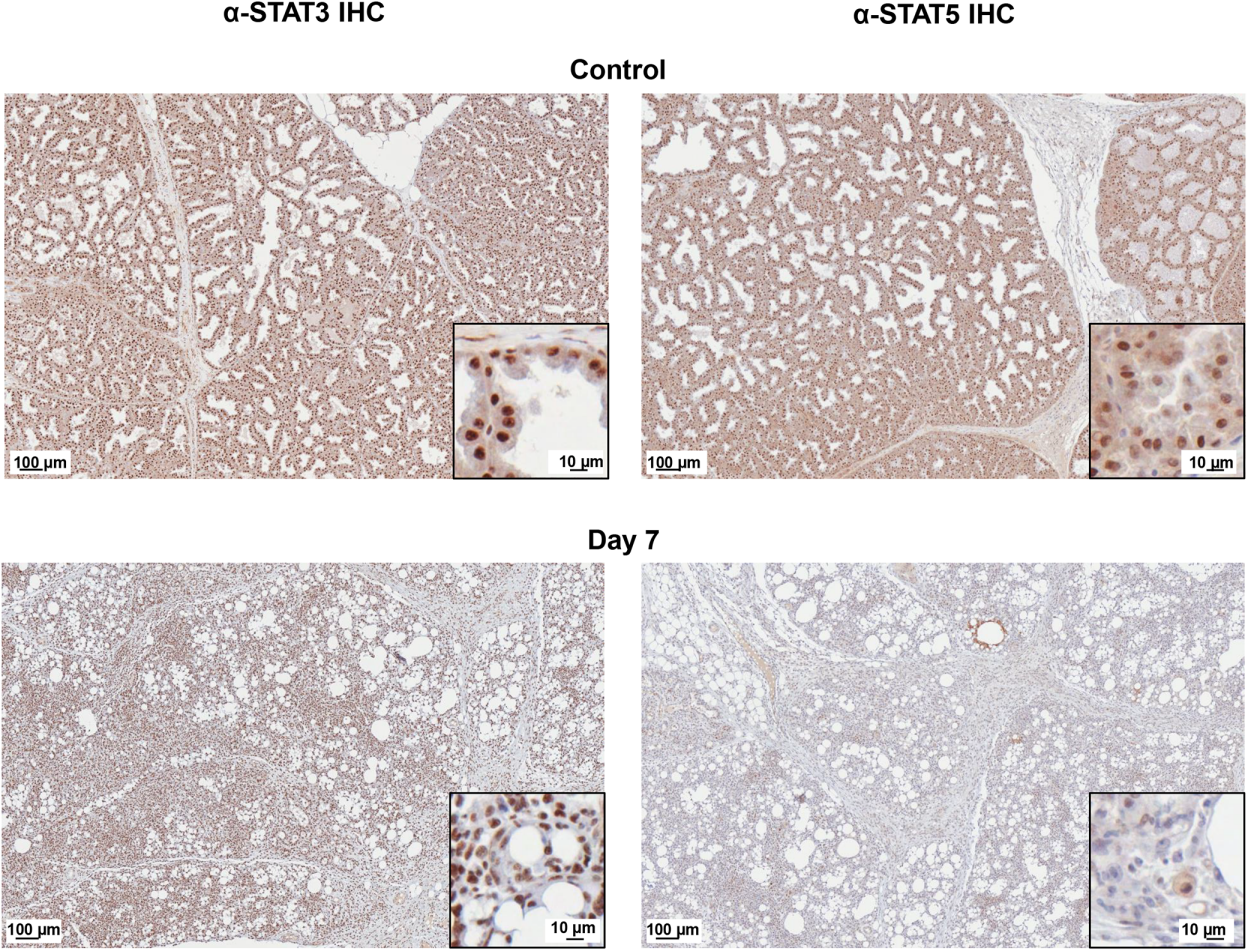
STAT5 protein is decreased and STAT3 protein is nuclear localized in H1N1+ Mammary Glands. STAT5 (right panels) and STAT3 (left panels) protein expression was visualized in Control and H1N1+MG on Day 7 post-infant-inoculation by IHC. IHC analysis of paraffin-embedded mammary sections from nursing-mothers of infected infants stained with STAT5 or STAT3 antibodies were visualized using an Aperio ScanScope XT, Leica Biosystems, Nußloch, Germany for high resolution scans. The inset picture shows a magnification of the scanned image to show cellular detail. Scale bars indicate 100 μm or 10 μm. The images show a representative of mammary glands collected from infant intranasal virus or mock inoculations (three inoculations each).

To further validate the gene expression results, we analyzed the protein expression of prominent regulators of gene networks discovered in our data analysis. IHC analysis of STAT3 expression in control and H1N1+ MGs (**Fig 8**, left panels) showed increased STAT3 protein expression in virus positive glands with specific increases within the nucleus and diminished cytoplasmic staining of the mammary gland cells (shown in the inset magnified picture). STAT5 protein was prominent in the control tissue but decreased in the H1N1+ samples (**Fig 8**, right panels). Together, our gene expression profiling and validation suggests influenza virus infection may regulate milk cessation, breast involution, and oncogenic microenvironment related gene networks.

Since H1N1+ MG had significant differentially regulated gene pathways, we also investigated the expression profiles of Bystander mammary glands to determine if these glands also had evidence of inflammation. We defined Bystander mammary glands to be mammary glands that were negative for virus by qRT-PCR but present on a mother who had H1N1+ MGs. It is not known if Bystander glands were previously virus infected and were able to clear the virus prior to collection and gene profiling. Parallel analyses of Bystander mammary glands (H1N1- at time of collection) partially recapitulated responses detected in H1N1+ MG (**S4 Fig**; **S4 Table**). Both H1N1+ and Bystander gland response signatures were characterized by upregulation of proliferation, remodeling, and immune response associated genes (**S5 Table**). Downregulation of sugar/lipid metabolism genes and STAT5A/B was also a shared feature of both H1N1+ and Bystander glands (**S4 Fig**; **S5 Table**). However, Bystander gland responses differed from H1N1+ MG as the *PPAR*/Hedgehog/Adipocytokine pathway was downregulated and the immune responses were less robust. Catabolism, apoptosis, and *TGF-β*/*p53*-associated gene responses characteristic of H1N1+ gland responses were also less pronounced in Bystander glands (**S4 Fig**).

We next sought to define the immune response regulation within the H1N1+ MGs compared to the canonical gene pathways that have been determined in influenza infected lungs. Clustal analysis of H1N1+ MGs alongside infected lungs revealed the common upregulation of antiviral response pathways but also tissue specific regulation of other immune genes (**Fig 7C**). Induction of genes typical of the influenza antiviral response [40] such as *CXCL10* and *CCL2* were seen Day 6/7 post-infant-inoculation in H1N1+ MG (**Fig 7C**). The immune response also differed in the mammary gland compared to the lung. The response in mammary tissue was characterized by decreased expression of lymphocyte-associated genes such as *CD3e*, *IL2Ra*, *CD4*, *AICDA* (Activation Induced Cytidine Deaminase) [41]; *IL4* and *IL1β* upregulation; and unchanged *IL6* expression (**Fig 7C**). These results suggest that influenza infected mammary glands are capable of producing an antiviral and immune response that is unique compared to the immune response mounted during infection in the respiratory tract.

### Mammary glands are directly susceptible to influenza virus infection leading to tissue pathogenesis and virus transmission to infants

**Fig 9:**
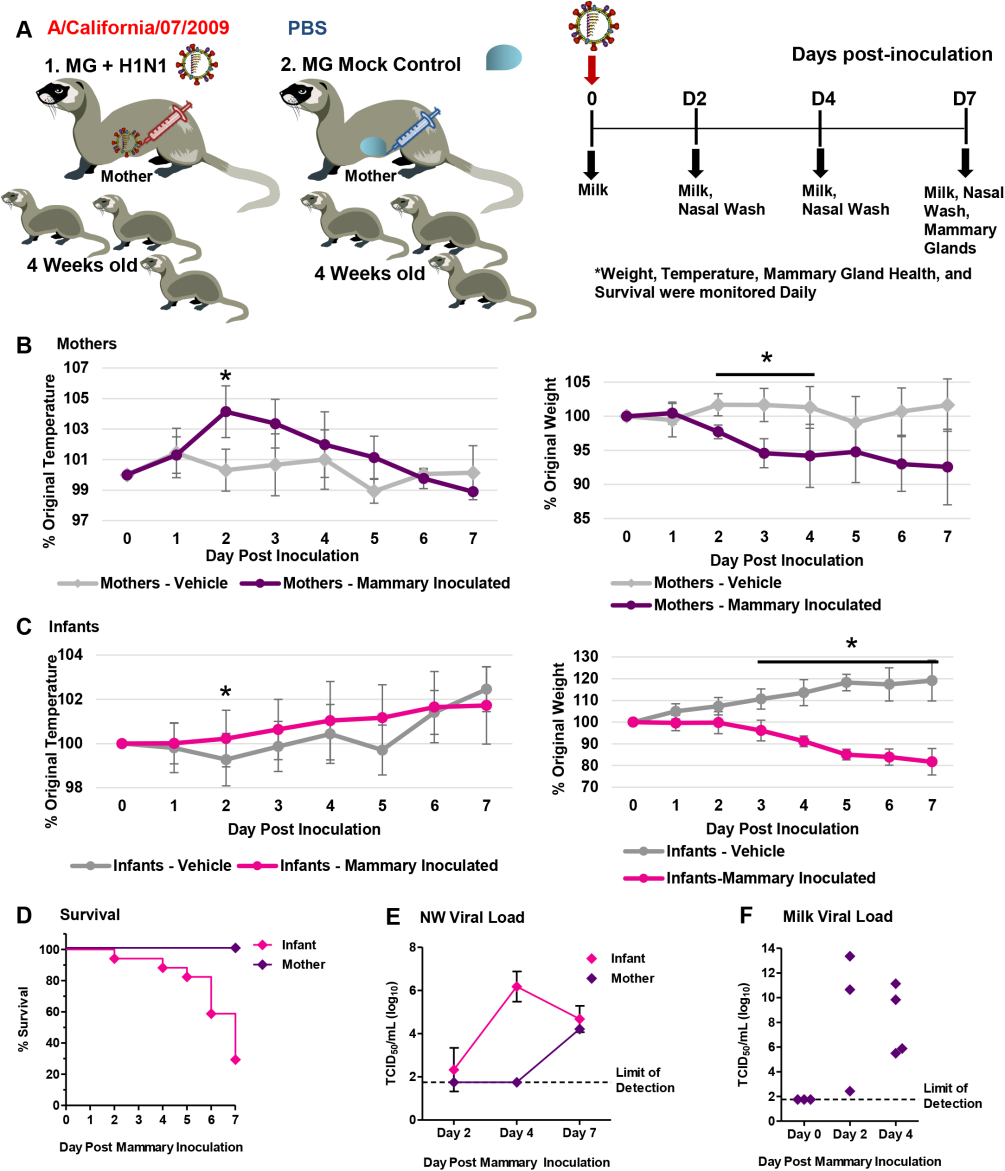
Influenza virus inoculation into active mammary glands of nursing-mother ferrets leads to respiratory infection, severe disease and mortality in infants. A schematic depicting the mammary gland inoculation design is shown (**A**). Temperature (left panel) and weights (right panel) of lactating mother ferrets following 2009 H1N1 (pink lines) or PBS (light grey lines) mammary inoculation (**B**). Temperature (left panel) and weights (right panel) of infants feeding from lactating mothers with 2009 H1N1 (pink lines) or PBS (light grey lines) inoculated mammary glands (**C**). Survival of mother and infant ferrets following mammary 2009 H1N1 virus inoculation (**D**). Live viral load determined from nasal wash in both infant and mothers (**E**), and live virus shedding from expressed milk (**F**) following mammary gland 2009 H1N1 inoculation. * Indicates a p-value less than 0.05 determined by ANOVA. Error bars indicate +/- SD. Results are the mean of or represent three independent mammary gland inoculation experiments (3 mammary inoculated mothers, 18 infants) and mock mammary inoculation controls (3 mammary mock inoculated mothers, 21 infants).

Above we determined that lactating mammary glands are able to host the influenza virus but it was not discerned if the mammary glands were directly susceptible to influenza infection. To determine if mammary glands are able to be infected with the influenza virus infection and act as a vessel of transmission, we inoculated active glands of lactating mother ferrets with Cal/07 (10^5^ EID_50_) or PBS through the lactiferous ducts (mammary gland inoculation experimental design, **Fig 9A**). This also allowed us to investigate the direct responses of influenza infection in the mammary gland as well as the role of the mammary gland in virus transmission.

Following direct inoculation with Cal/07, mothers and their infants developed significant disease characterized by temperature increases, weight loss, and infant mortality which was not observed in the vehicle inoculation groups (**Fig 9**). H1N1 mammary inoculated mothers developed a significant fever at 104% of original temperature Day 2 post-mammary inoculation not seen in the mock inoculation control mothers (**Fig 9B**, left panel). Virus inoculated mothers also lost between 7–10% of their original weight and did not recover by the end of study (**Fig 9B**, right panel). Infants feeding on virus inoculated mammary glands also had significant weight loss with a 30% survival rate by Day 7 post-mammary-inoculation (**Fig 9C**, right panel and **D**). Live virus, average 6 TCID_50_/ml (Log_10_), was detected in the NW of infants feeding from virus inoculated glands Day 4 post-mammary-inoculation (**Fig 9E**). Virus was detected in mother NW after detection in the infants (Day 7 pi). Live virus was present in expressed milk, Day 2 and Day 4 post-mammary-inoculation, between 3 and 13 TCID_50_/ml (Log_10_) there by confirming successful inoculation and infection (**Fig 9F**). As virus was detected in mother NW after in infant NW, these results suggested that infants developed respiratory infection directly from virus shed from the inoculated mammary gland.

**Fig 10:**
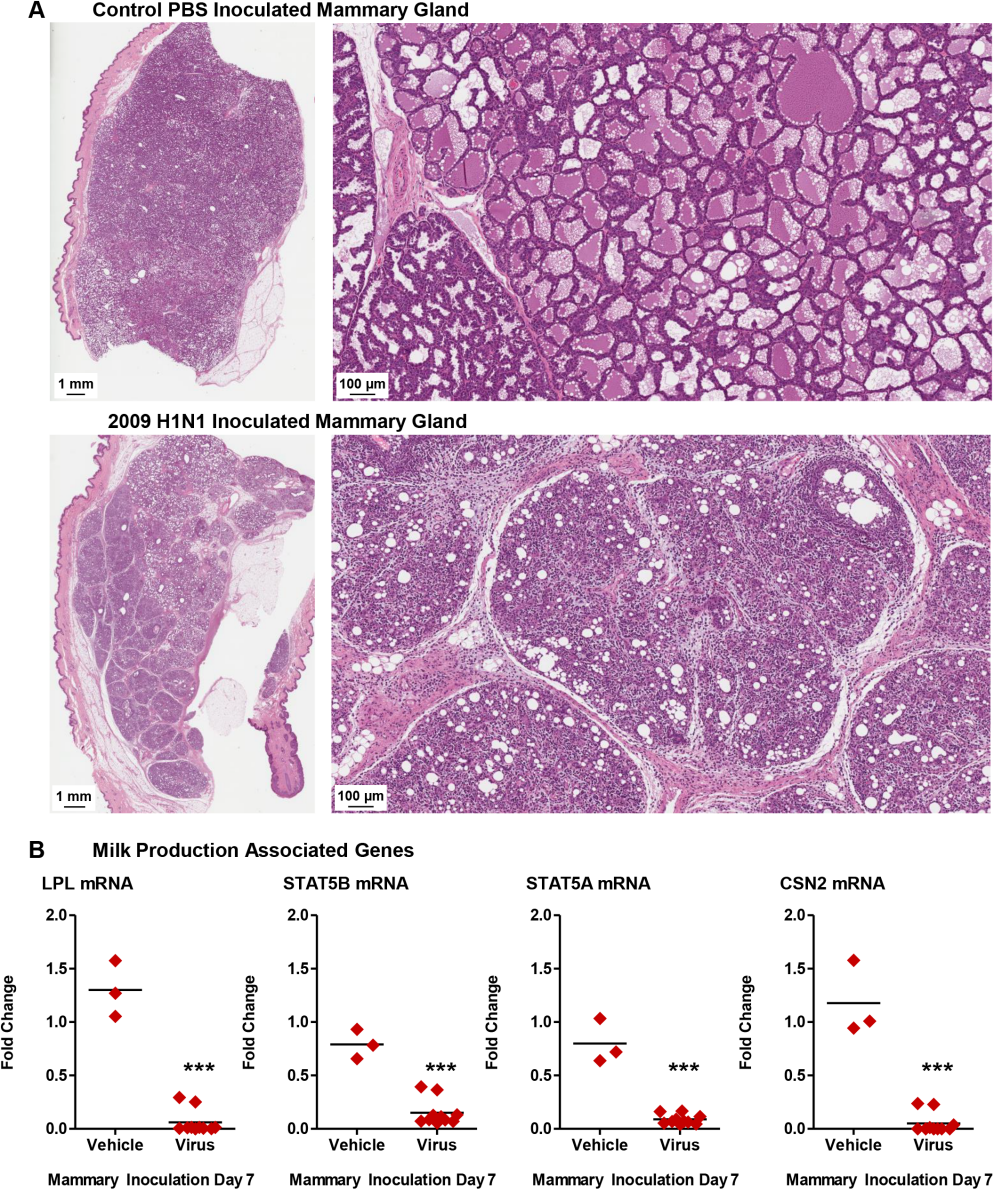
Influenza virus infection mammary glands causes milk cessation and the destruction of gland architecture. High resolution scan of Control and 2009 H1N1 inoculated mammary glands (H&E) (**A**). qRT-PCR profiles of milk production genes in 2009 H1N1 virus and vehicle inoculated mammary tissue (**B**). *** Indicates a p-value less than 0.001. Data was collected and analyzed from three independent mammary gland inoculation experiments (3 mammary inoculated mothers, 18 infants) and mock mammary inoculation controls (3 mammary mock inoculated mothers, 21 infants). Results presented show data or are representative of 3 mammary inoculations.

Mammary glands were stained with H&E to determine tissue architecture from direct inoculation of 2009 H1N1 and subsequent virus replication. Histopathology showed destruction of acini glandular architecture and absence of milk staining (**Fig 10A**). Gene expression profiling by qRT-PCR revealed Cal/07 directly inoculated mammary tissues had significant decreases in the milk production genes *STAT5A*, *STAT5B*, *LPL*, and *CSN2* (Ferret Primer Sequences **S3 Table**; **Fig 10B**). The β-casein protein was decreased in milk samples collected from inoculated tissue when it was able to be collected (**S5 Fig**). Taken together, direct mammary gland inoculation suggests influenza transmission is possible through breastfeeding and influenza virus infection in mammary tissue leads to pathogenesis and milk cessation.

**Fig 11:**
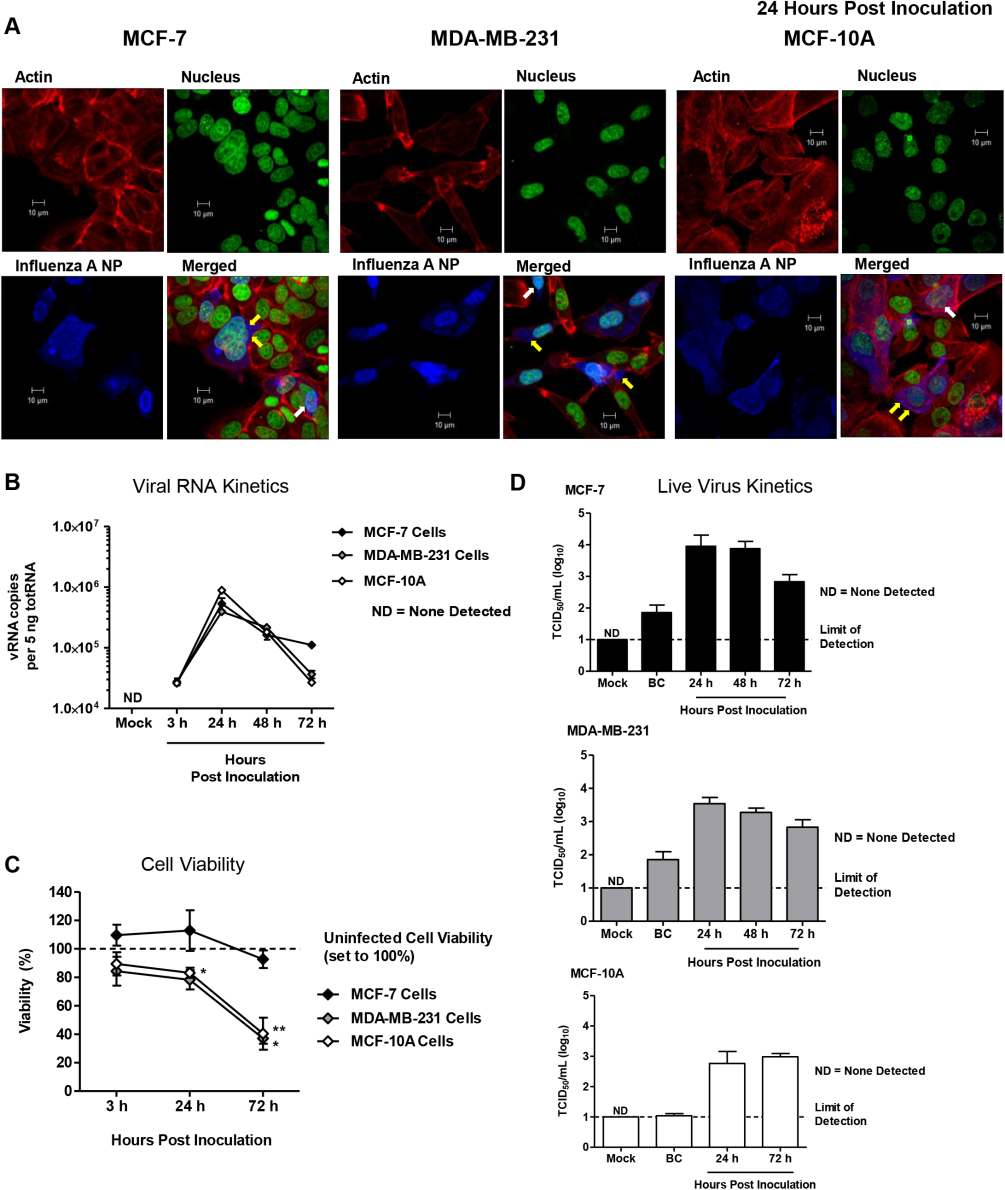
Human “normal” and adenocarcinoma mammary epithelial cells are susceptible and permissive to 2009 H1N1 infection *in vitro*. Mammary epithelial cells (MCF-7, MDA-MB-231, MCF-10A cells) inoculated at an MOI of 1 with A/Cal (H1N1) were fixed at 24 hours post-inoculation and stained for filamentous actin (red), DNA (green), and influenza A virus NP protein (blue), and imaged by confocal microscopy (**A**). Mammary epithelial cells (MCF-7, MDA-MB-231, MCF-10A cells) inoculated at an MOI of 1 with A/Cal (H1N1) were collected at 3, 24, 48, and 72 h post-inoculation for quantification of viral RNA segment 7 by qRT-PCR (**B**), determination of cell viability (**C**), and live virus quantification in supernatant (**D**). Confocal pictures are representative of three independent experiments. White arrows indicate nuclear localization of influenza NP protein. Yellow arrows show virus budding along the plasma membrane. BC, Baseline Control.

We next sought to determine if human breast cells were also susceptible and permissible to influenza virus infection. To this end we inoculated three cell lines of cultured human epithelial breast cells with Cal/07 strain (2009 H1N1) (in the absence of exogenous proteases) to visualize the virus life-cycle in inoculated cells, assess the viral kinetics, and determine cell viability post-inoculation (**Fig 11**). “Normal” non-tumorigenic (MCF-10A) and adenocarcinoma (MCF-7 and MCDA-MB-231) human epithelial breast cell lines were used to eliminate single cell type biases. To visualize the virus within the cell and determine virus subcellular localization, inoculated cells were stained for Influenza A Virus (IAV) NP (blue), nucleus (green), and actin filaments (red) (**Fig 11A**). Positive NP stain was used to determine if viral replication may be occurring within the nucleus. At 24 h post-inoculation influenza NP was detected in the nucleus (white arrows) of all three cell types, MCF-7, MDA-MB-231, and MCF-10A, indicated by co-localization of the nuclear stain (green) with NP staining (blue) (**Fig 11A**). Furthermore, marked punctate NP accumulation at the plasma membrane was observed in MCF-10A cells (yellow arrows) and to a smaller degree in the other cell types. vRNA was significantly increased in all cells types between 3 and 24 h post-inoculation by ~10 fold (**Fig 11B**). Cell viability analysis showed significant drops in cell viability of MCF-10A and MDA-MB-231 post-inoculation reaching ~35% viability at the 72 h time point compared to uninoculated cells at the same incubation (**Fig 11C**). To assess productive infection, live virus was quantified from collected supernatant at each time point. Live viral titers ranged between 3 and 4 TCID_50_/ml (Log_10_) for all cell types (**Fig 11D**). Baseline control (BC) wells had minimal or no detectable live virus. Together, these results suggest that the 2009 H1N1 virus was able to enter “normal” human breast cells leading to virus replication and productive infection.

## Discussion

Here we investigated influenza virus transmission within the mother-infant dyad using a novel ferret model. We found the influenza virus transmitted between nursing-mothers and infants in a bidirectional manner by respiratory and mammary transmission mechanisms. Transmission led to severe lower respiratory disease and mortality. Ferret mammary glands as well as human breast cells were both susceptible to infection and were able to replicate the virus efficiently suggesting mammary glands have the potential to function as a transmission vessel. Substantial changes in gene regulation were observed in H1N1 positive mammary tissue including induction of milk cessation genes and oncogenic pathways accompanying tissue pathogenesis. Influenza has caused high mortality in children (>280/season) and pregnant/postpartum women (~24%) during the influenza season in the United States [8,10] and our study may be used for future investigations of immune responses and therapeutic testing in these populations.

Infants and toddlers are infected with 8 to 10 respiratory viruses (such as RSV) as well as other common viruses (rotavirus, enteroviruses (EV71)) each year [24–27,42]. Our findings support investigation into the transmission dynamics of influenza and other childhood viruses during breastfeeding. Since retrograde flux of the mammary glands can allow liquid from infants to enter mammary ducts, viral transmission from the infected infants into the breast is possible via breastfeeding. The susceptibility of mammary glands to common pathogens may have both beneficial and deleterious long-term consequences which should be considered in future studies. As well, since vRNA was found in infant feces as well as in infant respiratory tracts, both respiratory and gastrointestinal introduction of the influenza virus are possible as routes of infection from mother to infant. Our gene expression profiling in virus positive mammary glands indicated induction of oncogenic pathways as well as immune responses [19–22]. Infection in mammary tissue may also evoke effective long-term adaptive immune responses. This may be a response conserved through evolution to serve as an advantage for future fecundity of that mother enabling protection by immune memory.

We found live virus was present in milk and within the mammary glands of mothers feeding 2009 H1N1 virus inoculated infants. Since live virus was shed in the milk, this suggested that infection may also be possible by contact with mother’s milk. Although we showed 2009 H1N1 virus presence in the mammary gland by four independent methods, immunohistochemistry, tissue viral load assay, milk viral load assay, and qRT-PCR, the kinetics of the virus expression in these assays were not always correlative. Since the milk vRNA and milk live viral load data were not aliquots of the same sample it is possible the amount of virus shed in milk may not be regular throughout the day or in each infection. RNA extraction from milk samples presented challenges due to the fat content of the milk which were not encountered in live viral load assessment of either milk or mammary gland tissue which may explain the higher live viral loads. As well, milk viral quantifications may differ from that of the mammary gland tissue viral quantification since virus in the milk may be diluted or concentrated dependent on the fullness of the mammary gland at the time of milking. Although these data did not directly correlate, the presence of virus in milk and within the mammary glands was a consistent finding.

To investigate the transmission potential through breastfeeding and the direct role of the mammary gland, we conducted direct virus inoculations to the mammary gland. By directly inoculating the mammary gland, we were able to eliminate the possibility of respiratory transmission in the initial portion of the experiment by isolating the infection within the mammary. Controlling the experiment through other means such as *the addition a non-lactating adult to the cage of mother and infants* brings ethical considerations as the presence of another adult would put the infant ferrets in danger. In our inoculation experiments, virus began shedding in milk Day 2 post-mammary-inoculation and was present in the infant NW between 2 and 4 days post-mammary-inoculation. Since virus was not detected in mother NW until later in the time course, this data suggested that the infants received the virus from infected mammary glands, not through the mother’s respiration. Virus transmitted from the mammary gland led to severe disease and infant mortality despite the atypical influenza transmission mode. Since infants are susceptible to respiratory tract infection by various microbes present in mammary glands/milk, such as bacteria and yeast [43–46], our findings showing respiratory infection from pathogens present in breast milk may have implications into possible sources of bacteria that may participate in influenza-bacterial co-infections in neonates. Moreover, influenza infection in mammary tissue may exacerbate or increase mastitis incidence in mothers and should be investigated in relation to resident and foreign microbial populations. Together, these results give insight into mechanisms which may contribute to influenza severity in infants and nursing-mothers.

The mammary gland is a unique organ which undergoes several structural and molecular transformations throughout the female’s life-cycle, i.e. puberty, pregnancy and postpartum. Pregnancy associated breast cancer (PABC) has been hypothesized to be a consequence of breast remodeling that occurs during pregnancy-lactation cycles and mammary involution [17,18]. We showed increases in oncogene pathways and involution programing in influenza positive glands. Breast involution is characterized by the presence of proteolytic cleavage, apoptosis, and leukocyte recruitment (i.e., macrophages) for secretory alveolar cell removal [17,47]. Our gene expression analysis implicated significant presence of M2 macrophages in influenza infected mammary glands by the upregulation of the M2 specific transcripts *CD163*, *CLEC7A*, *M5R1*, *CD209*, *LGMN*, and *MRC1*. M2 macrophages have a similar phenotype as tumor associated macrophages (TAM), since they regulate MMPs and support a microenvironment favorable for oncogenesis [47]. In agreement with this profile we showed increased *MMP1*, *MMP2*, *MMP8*, *MMP11* and *MMP14* in the H1N1+ MGs. We also found oncogenic collagen and cell attachment gene signatures upregulated in our data: *COL5A2*, *COL3A1*, *COL1A1*, *COL1A2*, and *COL4A6*, Integrins (*ITGB6*) and cell-matrix interaction proteins (*SPP1*)[17]. Several of these collagen genes have previously been shown to be associated with both cancer progression and involution in mouse models of mammary involution [17,48,49]. As further validation and discovery, future studies should investigate the full transcriptomics during influenza virus infection in active mammary glands of the ferret to eliminate any biases of the canine microarray. Although no structural or tissue pathological evidence for cancer progression was found in our studies possibly due to the short time frame of the study, clear tissue evidence of milk cessation was seen.

Viruses such as HPV have been shown or speculated to be the etiological agent of specific cancers [50] including breast cancer [19,51]. Breast cancer is the most common cancer affecting women worldwide [52] and viral nucleic acids have been found in breast tumors i.e., bovine leukemia virus (BLV) and mouse mammary tumor virus (MMTV) [19,51]. Viruses may instigate cancer progression through oncogenic viral proteins or by induction of chronic inflammation [19,53,54]. We found induction of genes typical of influenza antiviral responses (CXCL10) [40] as well as cancer associated genes upregulated in H1N1+ MG suggesting mammary glands. As our expression analysis showed the induction of a cancer-promoting microenvironment in mammary glands infected with the influenza virus, it is important to note that viruses that have been found to be directly oncogenic, such as HPV, have infectious cycles differing from that of the influenza virus [23]. Therefore we hypothesize if long-term biological networks are upregulated during influenza infection in breast tissue then those genetic changes would most likely be due to inflammation. The long-term implications of pathogen infection in breast tissue are not known but our data suggests influenza infection leads to short-term pathogenic genetic reprograming within mammary glands.

The CDC recommends continued breastfeeding in the event of infant influenza infection [55]. Although the importance of breastfeeding cannot be overstated [11,12], our findings may impact the management of influenza infection in the mother-infant dyad. Reports have shown most pregnant women with severe influenza infection did not receive the yearly influenza vaccine [10]. Our data reinforces the importance of seasonal influenza vaccination in pregnant and breastfeeding women. Our investigation also suggests that continued breastfeeding during mother or infant influenza infection could lead to breast infection, live virus shedding, and breast pathogenesis. Short-term milk expression by breast pumping with possible pasteurization may be an appropriate strategy for management of influenza infection in the mother-infant dyad. Previously, a guideline has been investigated for the management of influenza transmission in the mother-infant dyad. Since these guidelines had only considered the possibility of respiratory transmission, further investigation and guideline development may be important [56].

Although not much is known regarding viral transmission through breastfeeding, great efforts have been devoted to modeling mother-to-child HIV transmission due to the importance of breastfeeding in HIV high incidence countries [57]. HIV in breast milk exists in both cell-associated (leukocytes) and cell-free forms [34]. Our data suggests that the influenza virus infects glandular epithelial cells whereas HIV primarily targets leukocytes leading to systemic circulation. Our observation here implies novel dynamics of influenza transmission through breastfeeding is a mechanistically different mode of virus transmission in the mother-infant dyad and a formally unidentified system of immune response regulation within the mammary gland for the local production and direct delivery of pathogen specific antibodies. This suggests there is much to be understood regarding the mother-infant relationship and pathogen transmission. Recently, MERS-CoV has been speculated to be transmitted from camels to humans through consumption of camel milk [58]. Together these findings support a hypothesis that respiratory viruses may have the ability to also infect mammary tissue, including human breast cells, possibly due to a shared branched architecture and cellular structure of epithelial cells in lungs and mammary glands.

## Materials and Methods

### Ethics statement

All animal work was conducted in strict accordance with the Canadian Council of Animal Care (CCAC) guidelines. The protocol license number AUP 1031 was assigned by the Animal Care Committee of the University Health Network (UHN). UHN has certification with the Animals for Research Act including for the Ontario Ministry of Agriculture, Food and Rural Affairs, Permit Number: #0132–01 and #0132–05, and follows NIH guidelines (OLAW #A5408-01). The animal use protocol was approved by the UHN Animal Care Committee (ACC). All efforts were made to minimize animal suffering and infections and sample collections were performed under 5% isoflurane anesthesia.

### Influenza virus and animals

The 2009 H1N1 virus strain, A/California/07/2009 (Cal/07), in chicken egg allantoic fluid was provided by the Influenza Reagent Resource, Influenza Division, WHO Collaborating Center for Surveillance, Epidemiology and Control of Influenza, Centers for Disease Control and Prevention, Atlanta, GA, USA. TCID_50_ and EID_50_ determinations were done as previously described [59]. All virus work was performed in a BSL-2+ facility as previously described [29]. Four-week-old-male and female ferrets, female Jill ferrets (aged 5 months to 1 year) and male ferrets 8 months old (adult) were bred in an on-site SPF ferret colony (University Health Network, Toronto, ON, Canada). Ferrets were shown to be seronegative by haemagglutination inhibition (HI) assay against currently circulating influenza A and B strains before infection. Infant ferrets were housed in litters with nursing-mother ferrets. Litters were housed according to UHN ferret colony specifications.

### Influenza inoculation, mammary gland influenza inoculation, and ferret monitoring

Ferrets were maintained and monitored in litters or as previously described (adults) [29,32]. Briefly, prior to inoculation, 4-week-old nursing ferret litters were randomly selected and housed in appropriate litter boxes contained in bioclean portable laminar-flow clean-room enclosures (Lab Products, Seaford, DE, USA) in the BSL-2+ animal holding area. Baseline body temperature and weight were measured on Day -1 and 0 for each animal. Temperatures were measured by using a subcutaneous implantable temperature transponder (BioMedic Data Systems, Inc., Seaford, DE, USA) for mothers and adults. Infant temperature was measured rectally by DT-610B Single Input K-Type Thermometer (ATP Instrumentation, Leicestershire, United Kingdom).

#### Inoculations

On the day of inoculation, each ferret was anesthetized and inoculated with 1 ml of Cal/07 inoculum (0.5ml in each nostril) for adults or 200 μl (100 μl in each nostril) for infants both at dose of 10^5^ 50% egg infectious dosage (EID_50_). For each infection experiment three litters at 4-weeks postpartum were randomly chosen for each experimental group (Inoculation or mock control). Each litter included a mother and her infants from a single birthing litter which ranged between 5 and 11 kits depending on the litter. Infection replications were performed simultaneously as well as independently. Data presented represents a minimum of 3 litters with the animal numbers used for data presentation specified in the figure legends where needed.

#### Mammary gland inoculations

For mammary gland inoculations, mother ferrets of 4 weeks postpartum were anesthetized using isoflurane as previously described for adult ferrets [29]. The mammary glands were warmed with a warm press to express milk. The inoculation site was then cleaned with ethanol. A/Cal H1N1 virus (10^5^ EID_50_) or PBS control at 125 μl of inoculum was injected by guided ultrasound into the lactiferous duct of the active mammary gland under the supervision of a veterinarian. Half of the active mammary glands were inoculated in a single animal at one time. Mammary glands chosen for inoculation were staggered with mammary glands that were left unaltered to ensure continued feeding by the infant ferrets. Following inoculation, the mother was observed until she was fully awake in the absence of the infant ferrets. The mammary glands were cleaned with ethanol prior to infant ferret reintroduction.

#### Clinical monitoring

Clinical signs (body temperature, body weight, activity score, symptoms of nasal discharge, sneezing and mammary gland health) were observed daily for 14 days post-infant-inoculation or post-mother-inoculation. Mother ferrets can have a variable number of active mammary glands per pregnancy/postpartum (~5–8). Therefore, a variable number of mammary glands were assessed for viral loads per time point per animal. Mothers and infants were observed twice daily following mammary gland inoculations. Animals were examined at the same time each day for consistency. Nasal discharge and sneezing was observed but confident readings were not able to be recorded due to the small size of the infant ferrets. As well, activity score were not able to be accurately assessed in the infant ferrets due to their low normal activity levels. Mammary glands were observed twice daily following virus inoculation for signs of mastitis (redness, firm tissue and heat) or adverse reaction to injection (hemorrhage, swelling). No signs of adverse reactions or mastitis were noted from inoculation with virus or PBS control.

### Sample collection (nasal wash, milk, feces, and tissues)

Nasal washes from the inoculated ferrets were collected on specified days post-inoculation in nasal wash buffer (1% BSA and 100 U/ml penicillin, 100 μg/ml streptomycin in PBS) as previously described [29]. One ml (adults) or 500 μl (infants) of nasal wash buffer was used for each collection. Penicillin and streptomycin were obtained from Invitrogen Canada (Burlington, ON, Canada) and BSA was from Wisent Inc. (Saint-Bruno, QC, Canada). For milk collection, mammary glands were cleaned and a warm press was applied to increase the easy of milking the gland. Milk was collected and pooled from all mammary glands of one mother. Milk for vRNA and live viral load assessments were collected at different milking sessions. Milk was stored in RLT Buffer or directly frozen at -80^°^C. Tracheas, lungs, mammary glands, and nipples were collected on specified days post-inoculation from the euthanized ferrets. Prior to mammary gland collection, nipples were externally cleaned with ethanol and then the nipples were removed. Internal mammary gland tissue was harvested subsequent to nipple removal. Mother ferrets can have a variable number of active mammary glands per pregnancy/postpartum (~5–8). Therefore, a variable number of mammary glands was assessed for viral loads per time point. As many mammary glands were run as possible to acquire the maximum amount of data. Although the number of glands was variable, the number of mother ferrets was always three ferrets per time point. Feces from infant ferrets were collected at the time of anal temperature collection. Each feces was placed directly in RLT buffer or placed directly at -80^°^C from the animals’ anus to avoid surface contamination. Samples were stored in -80^°^C before processing for viral loads or RNA. Samples for RNA were either placed in RNA later (milk and feces) or homogenized in TRIzol (Tissues). Sample aliquots for histopathology were stored in formalin until processing.

### Determination of viral load

Viral replication in the upper, middle, and lower respiratory tract and mammary tissues were assessed by endpoint titration of nasal washes or homogenized tissue samples from the inoculated ferrets in MDCK cells (TCID_50_) using haemagglutination as the readout for positive wells as previously described[29]. Briefly, nasal washes were diluted 1:10 in vDMEM (Dulbecco's modified Eagle's medium containing 1% BSA, 25mM glucose, 1mM sodium pyruvate, 4mM glutamine, 100 U/ml penicillin, 100 μg/ml streptomycin, 50 μg/ml gentamycin, and 1 μg/ml TPCK-Trypsin) followed by the half log serial dilution in quadruplicate with vDMEM on MDCK cells in 96-well flat-bottom plates (SARSTEDT, Inc., Saint-Leonard, QC, Canada). Tissues were processed similarly and homogenized 1:10 (w/v) in sDMEM (vDMEM excluding 1% BSA) on ice and centrifuged before titration of the homogenates on MDCK cells. Before inoculation, MDCK cells were maintained in the log-phase at low-passage numbers and grown in cDMEM (DMEM containing 10% fetal bovine serum, 25 mM glucose, 1 mM sodium pyruvate, 6 mM glutamine, 1 mM non-essential amino acids, 100 U/ml penicillin, and 100 μg/ml streptomycin). The day before viral sample titration, 2x10^4^ MDCK cells were seeded into each well to reach 95% confluence on the next day. After a two-hour incubation of titrated viral samples with MDCK cells at 37^°^C, 5% CO_2_, samples were aspirated and replaced with fresh vDMEM followed by a six-day incubation at 37^°^C, 5% CO_2_. On Day 6 post-incubation, supernatants were examined for presence of virus by haemagglutination of 0.5% turkey erythrocytes (Lampire Biological Laboratories, Pipersville, PA, USA). The viral titers were determined as the reciprocal of the dilution resulting in 50% HA positivity in the unit of TCID_50_/ml represented in log_10_ base. Viral titers in cell culture supernatants were also determined to investigate capacity of mammary epithelial cells to produce infectious virus in vitro. Cell culture supernatants were loaded directly and serially diluted as described above. Assay upper and lower limits of detection depended on sample availability and/or expected viral titer and varied between experiment types. Lower limits of detection are indicated in all viral load figures by a dashed line. Upper limits of detection were 10^6.75^ TCID_50_/ml for mammary epithelial cell supernatant assessments, 10^7.75^ and 10^13.75^ for milk assessments in **Fig 5D** and **Fig 9F**, respectively, and 10^7.25^ for all nasal wash and tissue assessments except for **S1C Fig** (10^7.75^). Cell culture reagents were obtained from Invitrogen Canada or Wisent except for TPCK-Trypsin (Sigma-Aldrich Canada Ltd., Oakville, ON, Canada).

### Histopathology and immunohistochemistry

Animals were euthanized on appropriate days and the lung or mammary gland tissues were harvested, perfused and fixed in formalin followed by paraffin embedding and sectioning as described [29]. Tissue slides were then stained with hematoxylin and eosin for histopathology assessment. Nursing-mothers of mock-inoculated 4-week-old infants were used for health and age control. Immunostaining was performed using a polyclonal goat anti-influenza antibody (1:2000) (Abcam (ab20841), Cambridge, United Kingdom). STAT3 protein was detected with a rabbit polyclonal anti-STAT3 (LSBio, Cat # LS-B4693) using CIT6 Antigen Retrieval at 1:300 primary antibody dilution for 1 hour incubation. STAT5 protein was detected with a rabbit polyclonal anti-STAT5 (Abcam, Cat # ab68465) using TE9 Antigen Retrieval at a concentration of 1:100 primary antibody dilution and a 1 hour incubation. High resolution scans were performed using an Aperio ScanScope XT, Leica Biosystems, Nußloch, Germany.

### Western blot analysis

Milk was expressed from the mammary glands of lactating mother ferrets. Ten μg of milk was load per lane and proteins were separated by SDS-PAGE and transferred to PVDF membrane. To visualize beta-casein protein, the membrane was blotted with 1:500 rabbit polyclonal anti-bovine beta-casein (Cat# 251309, Abbiotec) and followed with 1:2000 anti-rabbit IgG-HRP (Santa Cruz sc-2030). To ensure equal loading of each sample, a protein estimation was conducted using a Pierce BCA Protein Assay Kit (Life Technologies). Proteins were detected with a chemiluminescence protocol and exposed. Images were exposed to Thermo Scientific CL-XPosure Film (Waltham, MA, USA) and developed using a Konica Minolta Model SRX-101A (Tokyo, Japan) machine.

### RNA extraction and quantitative Real-Time PCR (qRT-PCR)

Collected lung and mammary tissue was placed directly in TRIzol reagent (Life Technologies, Burlington, Ontario) for immediate homogenization and subsequently processed according to the manufacturer’s instructions as previously done [32]. Milk RNA was extracted using an RNEasy Kit (Qiagen). Fecal RNA was extract using QIAshredder (Qiagen) before subsequent processing by RNEasy Kit (Qiagen, Hilden, Germany). Total RNA was converted into cDNA using ImProm-II reverse transcription system (Promega Inc., Madison, WI, USA) followed by qRT-PCR. qRT-PCR was performed on the ABI Prism 7900HT system (Applied Biosystems, Foster City, CA, USA). Blood was collected in PAXgene Blood RNA tubes followed by extraction using a PAXgene Blood RNA Kit (PreAnalytiX, Hombrechtikon, Switzerland), per the manufacturer’s instructions. Quadruplicate PCR reactions were run for specified target genes, each containing 4 μl 1.25 ng/μl cDNA, 0.5 μl 4μM forward and reverse primers, and 5 μl SYBR green PCR master mix (Applied Biosystems Inc., Carlsbad, CA, USA). PCR reactions were run at an annealing temperature of 60^°^C for 35 cycles for blood, tissue, and cell lysates. PCR reactions for milk and fecal RNA were done at 60^°^C for 40 cycles due to the difficulty of extracting RNA from milk and feces. Applied Biosystems Sequence Detection Systems Version 2.4 software was used for raw data collection (Applied Biosystems, Foster City, CA, USA). Target gene expression levels were normalized to house-keeping gene β-actin, and quantified by the relative standard curve method.

### Microarray gene expression analysis

RNA extracted from mammary glands of nursing-mothers (2009 H1N1 and mock inoculated infants) and influenza infected adult lungs were subjected to gene expression analysis by microarray. Mammary glands were determined to be virus infected glands if qRT-PCR found vRNA >10 copies per 5 ng of RNA and histological evidence of virus infection. Mammary glands were determined to be Bystander glands if vRNA was undetectable by qRT-PCR. Total cellular RNA from tissues was collected and purified using TRIzol reagent and methods, as described above. Complementary RNA (cRNA) was generated using 3’ IVT Express Kit (Affymetrix, Santa Clara, CA, USA) and microarray analysis was performed using Affymetrix GeneChip Canine Genome 2.0 Array (Affymetrix, Santa Clara, CA, USA), as previously described [40]. We have previously used and validated canine arrays for the investigation of ferret global gene expression profiling [60–64]. We demonstrated high levels of homology (average of 89% identity) between 30 canine and ferret nucleotide sequences [60]. Data sets for H1N1+ mammary glands, bystander mammary glands, and lungs were pre-processed independently with quantile normalization, variance stabilization, and log_2_ transformation. For genes represented by multiple probes on the array, the probe with highest total signal in H1N1+ mammary gland and lung datasets were selected for analysis. Two parallel analyses were applied A H1N1+ and bystander mammary gland datasets. Global clustering analyses were performed by one-way hierarchical clustering (Pearson’s correlation) of all significantly differentially regulated genes (p-value<0.05 Student’s t-test unpaired, equal variances, fold change ≥ |1.5| fold) and functional annotation of generated clusters. Global pathway gene enrichment analyses were performed by functional annotation of genes exhibiting significant changes in expression at either Days 3/4 or 6/7 in either dataset, using the KEGG Pathway database. DAVID Bioinformatics Resource v6.7, described previously [40] was used for functional annotation and enrichment score profiling. Clustergrams in **Fig 7B** were manually selected as representative genes for signaling pathways and gene networks prominently represented in global analyses. Genes included in clustergrams were identified as significantly differentially regulated members of related KEGG-defined signaling pathways with the exception of Macrophage and Milk Production clustergrams which were generated by manual selection of representative genes. Full clustergrams of all significantly differentially regulated genes for each KEGG-signaling pathway identified in **Fig 7**are included in **S2 Fig**

### Data availability

Accompanying data can be found in the Supplementary Materials. Raw (.CEL files) and normalized microarray data were compiled in accordance with Minimum Information About a Microarray Experiment (MIAME) guidelines and uploaded to the public data repository Gene Expression Omnibus (GEO) for public dissemination (www.ncbi.nlm.nih.gov/geo/). These datasets can be found under the accession numbers GSE63082 (mammary gland), GSE63083 (lung) and GSE63084 (both) at http://www.ncbi.nlm.nih.gov/geo/query/acc.cgi?acc=GSE63084.

### In vitro analysis and confocal microscopy

In vitro studies were performed similarly as reported previously [65]. MCF-10A, MFC-7, and MDA-MB-231 cells were purchased from ATCC. The cells were seeded at 50,000 cells/well in 6 well plates for RNA and viral load analysis. Cells were seeded at 75,000 cells per well in Nunc Lab-Tek II Chamber Slide System, 2 well glass slides (Thermo Scientific) for confocal microscopy. Cells were inoculated with A/Cal (H1N1) at a dose of 10^4^ (ELISA assay calculation for MO1 = 1) TCID_50_ (determined by MDCK titration) per 10^4^ cells (10^6.5^ TCID_50_ HA assay calculation). For confocal, cells were fixed in 4% PFA in PBS at 24 hours post-inoculation and stained for filamentous actin using Phalloidin (Molecular Probes), DNA using SYTOX green (Molecular Probes), and influenza A virus NP protein using a mouse monoclonal [AA5H] to Influenza A Virus Nucleoprotein (Abcam; ab20343) at 1:100 for 60 min. This was followed by staining with Alexa Fluor 647 Donkey Anti-Mouse secondary (Jackson ImmunoResearch). Prior to staining cells were permeablized using Triton X-100 in PBS. Slides were imaged by confocal microscopy using a Zeiss LSM 710 NLO, using objective C-Apo 63x/1.4 NA (oil). For RNA and viral load, cell lysates and supernatants were collected at 3, 24, 48, and 72 h post-inoculation for RNA extraction and live viral load quantification, respectively. The baseline control was determined by incubating the inoculum in assay wells without cells for 72 h. to assess virus decay and viral attachment to the wells. qRT-PCR and viral load were performed as described in above methods section.

### Statistical analyses

Unpaired, unequal variance, two-tail Student’s *t*-test or one-way ANOVAs were conducted. The Log-rank (Mantel-Cox) Test was used to determine significant differences in animal survival. A *p* value of ≤ 0.05 was considered statistically significant.

## Supporting Information

S1 Table Mean weight and temperature readings of mothers of mock infected infants. (PDF)

S2 Table Gene enrichment scores. (PDF)

S3 Table qRT-PCR primer sequences. (PDF)

S4 Table Functional annotation analysis for bystander mammary glands. (PDF)

S5 Table Functional annotation analysis for bystander / H1N1+ mammary gland comparison. (PDF)

S1 Fig Nasal wash viral loads of intranasal inoculated infant ferrets and nursing-mothers over a 10 day time course. Nasal washes from infant ferrets and their feeding mothers were collected on Day 3, 7 and 10 post-infant inoculation (**A**) or post-mother inoculation (**B**) with 2009 H1N1 A/Cal (10^5^ EID_50_). Live viral loads were assessed by titration on MDCK cells for active viral shedding throughout the time course. Adult ferrets were pair-housed and one ferret was intranasally inoculated with 2009 H1N1 A/Cal (10^5^ EID_50_) (**C**). Viral loads were determined for collected NW from both inoculated and naïve cage mate ferrets.
(PDF)

S2 Fig Pathways differentially regulated in 2009 H1N1 positive mammary glands. (**A**) Clustergram of differentially regulated genes involved in Jak-STAT signaling. One-way hierarchical clustering analysis (Pearson’s correlation) was performed on all probes of significantly differentially regulated genes (≥ |1.5| fold-change in expression, p-value < 0.05) classified as members of Kyoto Encyclopedia of Genes and Genomes (KEGG) Pathway database cfa04630 (left panels). Downregulated genes are in blue, upregulated genes in red. Visualization of Jak-STAT signaling pathway by Ingenuity Pathway Analysis (IPA) (right panels). Gene expression values from D6/7 mammary glands (average of n = 3) were overlaid on IPA pathway entry Jak/STAT Signaling (blue: downregulated genes; red: upregulated genes). (**B**) Clustergram of differentially regulated genes involved in TGF-β signaling. One-way hierarchical clustering analysis (Pearson’s correlation) was performed on all probes of significantly differentially regulated genes (≥ |1.5| fold-change in expression, p-value < 0.05) classified as members of Kyoto Encyclopedia of Genes and Genomes (KEGG) Pathway database (left panels) cfa04350. Downregulated genes are in blue, upregulated genes in red. Visualization of TGF-β signaling pathway by Ingenuity Pathway Analysis (IPA) (right panels). Gene expression values from D6/7 mammary glands (average of n = 3) were overlaid on IPA pathway entry TGF-β Signaling (blue: downregulated genes; red: upregulated genes). (**C**) Clustergram of differentially regulated genes involved in Wnt-β-Catenin signaling. One-way hierarchical clustering analysis (Pearson’s correlation) was performed on all probes of significantly differentially regulated genes (≥ |1.5| fold-change in expression, p-value < 0.05) classified as members of Kyoto Encyclopedia of Genes and Genomes (KEGG) Pathway database (left panels) cfa04310. Downregulated genes are in blue, upregulated genes in red. Visualization of Wnt signaling pathway by Ingenuity Pathway Analysis (IPA) (right panels). Gene expression values from D6/7 mammary glands (average of n = 3) were overlaid on IPA pathway entry Wnt Signaling (blue: downregulated genes; red: upregulated genes). (**D**) Clustergram of differentially regulated genes involved in p53 signaling. One-way hierarchical clustering analysis (Pearson’s correlation) was performed on all probes of significantly differentially regulated genes (≥ |1.5| fold-change in expression, p-value < 0.05) classified as members of Kyoto Encyclopedia of Genes and Genomes (KEGG) Pathway database (left panels) cfa04115. Downregulated genes are in blue, upregulated genes in red. Visualization of p53 signaling pathway by Ingenuity Pathway Analysis (IPA) (right panels). Gene expression values from D6/7 mammary glands (average of n = 3) were overlaid on IPA pathway entry p53 Signaling (blue: downregulated genes; red: upregulated genes). (**E**) Clustergram of differentially regulated genes involved in Neutrophin/RTK signaling. One-way hierarchical clustering analysis (Pearson’s correlation) was performed on all probes of significantly differentially regulated genes (≥ |1.5| fold-change in expression, p-value < 0.05) classified as members of Kyoto Encyclopedia of Genes and Genomes (KEGG) Pathway database (left panels) cfa04722. Downregulated genes are in blue, upregulated genes in red. Visualization of Neutrophin/RTK signaling pathway by Ingenuity Pathway Analysis (IPA) (right panels). Gene expression values from D6/7 mammary glands (average of n = 3) were overlaid on IPA pathway entry Neutrophin/RTK Signaling (blue: downregulated genes; red: upregulated genes). (**F**) Clustergram of differentially regulated genes involved in PPAR signaling. One-way hierarchical clustering analysis (Pearson’s correlation) was performed on all probes of significantly differentially regulated genes (≥ |1.5| fold-change in expression, p-value < 0.05) classified as members of Kyoto Encyclopedia of Genes and Genomes (KEGG) Pathway database (left panels) cfa03320. Downregulated genes are in blue, upregulated genes in red. Visualization of PPAR signaling pathway by Ingenuity Pathway Analysis (IPA) (right panels). Gene expression values from D6/7 mammary glands (average of n = 3) were overlaid on IPA pathway entry PPAR Signaling (blue: downregulated genes; red: upregulated genes). (**G**) Clustergram of differentially regulated genes involved in Toll-Like Receptor signaling. One-way hierarchical clustering analysis (Pearson’s correlation) was performed on all probes of significantly differentially regulated genes (≥ |1.5| fold-change in expression, p-value < 0.05) classified as members of Kyoto Encyclopedia of Genes and Genomes (KEGG) Pathway database (left panels) cfa04620. Downregulated genes are in blue, upregulated genes in red. Visualization of Toll-Like Receptor Signaling Pathways by Ingenuity Pathway Analysis (IPA) (right panels). Gene expression values from D6/7 mammary glands (average of n = 3) were overlaid on IPA pathway entry Toll-Like Receptor Signaling (blue: downregulated genes; red: upregulated genes). (**H**) Clustergram of differentially regulated gene signaling in Cancer related pathways. One-way hierarchical clustering analysis (Pearson’s correlation) was performed on all probes of significantly differentially regulated genes (≥ |1.5| fold-change in expression, p-value < 0.05) classified as members of Kyoto Encyclopedia of Genes and Genomes (KEGG) Pathway database (left panels) cfa05200. Downregulated genes are in blue, upregulated genes in red. Visualization of Molecular Mechanisms of Cancer signaling pathway by Ingenuity Pathway Analysis (IPA) (right panels). Gene expression values from D6/7 mammary glands (average of n = 3) were overlaid on IPA pathway entry Molecular Mechanisms of Cancer Signaling (blue: downregulated genes; red: upregulated genes). (**I**) Clustergram of differentially regulated genes involved in Cell Cycle signaling. One-way hierarchical clustering analysis (Pearson’s correlation) was performed on all probes of significantly differentially regulated genes (≥ |1.5| fold-change in expression, p-value < 0.05) classified as members of Kyoto Encyclopedia of Genes and Genomes (KEGG) Pathway database (left panels) cfa04110. Downregulated genes are in blue, upregulated genes in red. Visualization of Cyclins and Cell Cycle signaling pathway regulation by Ingenuity Pathway Analysis (IPA) (right panels). Gene expression values from D6/7 mammary glands (average of n = 3) were overlaid on IPA pathway entry Cyclins and Cell Cycle Signaling (blue: downregulated genes; red: upregulated genes). (**J**) Clustergram of differentially regulated genes involved in Cell Attachment signaling. One-way hierarchical clustering analysis (Pearson’s correlation) was performed on all probes of significantly differentially regulated genes (≥ |1.5| fold-change in expression, p-value < 0.05) classified as members of Kyoto Encyclopedia of Genes and Genomes (KEGG) Pathway database (left panels) cfa04510 (Focal Adhesion signaling) and cfa04520 (Adherens Junction signaling). Downregulated genes are in blue, upregulated genes in red. Visualization of Epithelial Adherens Junction Signaling pathway by Ingenuity Pathway Analysis (IPA) (right panels). Gene expression values from D6/7 mammary glands (average of n = 3) were overlaid on IPA pathway entry Epithelial Adherens Junction Signaling (blue: downregulated genes; red: upregulated genes). (**K**) IPA Symbol Key taken from the IPA user manual.
(PDF)

S3 Fig Real-Time RT-PCR analysis of target genes regulated during 2009 H1N1 infection in mammary glands. Real-Time PCR was conducted on RNA extracted from 2009 H1N1 ferret mother mammary gland for genes representing Milk Production, Cancer-Associated, and Immune Response pathways. Mammary glands were collected from nursing mothers on Day 3/4 or Day 6/7 post-infant infection and RNA was extracted.
(PDF)

S4 Fig Bystander mammary glands partially recapitulate gene signature of 2009 H1N1 positive glands. Global clustering analysis of all significantly differentially regulated genes (p-value<0.05, fold change ≥1.5 fold) at Day 3/4 and/or Day 7 in bystander mammary glands; total number of genes: 6821. Hallmark functional groups of each cluster are indicated (**Ai**). Gene enrichment score profiles for all KEGG-defined signaling pathways which had exhibited significant enrichment among either upregulated or downregulated gene subsets at Days 3/4 or Day 7 (see **S4 Table** for more detail) (**Aii**). Clustergrams of significantly differentially regulated genes associated with immune processes (**Aiii**). Comparative transcriptomic analysis of H1N1+ and bystander mammary glands. The number of significantly differentially regulated genes in H1N1+ glands only, bystander glands only, or both are indicated by Venn diagram and the most prominent functional groups for each gene subset are described (see **S5 Table** for more detail) (**B**). Upregulated genes are labeled red and downregulated genes are labeled blue throughout. Samples were collected and analyzed from six independent litter experiments (n = 3/time-point).
(PDF)

S5 Fig Beta-Casein protein is deceased in milk samples from mammary glands infection with 2009 H1N1. Lactating ferret mothers were inoculated intramammary gland with Cal/07 at 10^5^ EID_50_. Expressed milk was electrophoresed by SDS-PAGE and analyzed by western blot with a beta-casein antibody.
(PDF)
