# Supplementary material for: Influenza Transmission in the Mother-Infant Dyad Leads to Severe Disease, Mammary Gland Infection, and Pathogenesis by Regulating Host Responses
Source: PLoS Pathog. 2015 Oct 8;11(10):e1005173. doi: 10.1371/journal.ppat.1005173 (PMC4598190; doi:10.1371/journal.ppat.1005173)
Supplement: S1 Table — (PDF) [file ppat.1005173.s001.pdf]

S1 Table. Mean Weight and Temperature Readings of Mothers of Mock Infected Infants

	Baseline	1	2	3	4	5	6	7	8	9	10	11	12	13	14
Temperature	100	99.78671	100.4604	101.0602	100.9083	99.78129	100.3032	100.7288	99.6966	100.6433	101.3316	100.9896	99.01317	99.79082	100.4752
Weight	100	101.4476	100.5101	100.2057	98.70447	98.6431	99.88197	101.1513	102.0838	101.7276	101.4811	101.6606	98.86033	98.86033	99.87296
