# Supplementary material for: Influenza Transmission in the Mother-Infant Dyad Leads to Severe Disease, Mammary Gland Infection, and Pathogenesis by Regulating Host Responses
Source: PLoS Pathog. 2015 Oct 8;11(10):e1005173. doi: 10.1371/journal.ppat.1005173 (PMC4598190; doi:10.1371/journal.ppat.1005173)
Supplement: S2 Table — (PDF) [file ppat.1005173.s002.pdf]

S2 Table. Gene Enrichment Scores

Upregulated Genes

Day 3/4; ↑

Gene Classification Group	Score ($-log_{10}$)
**Splicesome** KEGG (cfa03040)	4.28
**Ubiquitin Mediated Proteolysis** KEGG (cfa04120)	2.12
**RNA Degradation** KEGG (cfa03018)	1.96
**Neutrophin Signaling Pathway** KEGG (cfa04722)	1.67
**Pathways in Cancer** KEGG (cfa05200)	1.57
**Adherens Junction** KEGG (cfa04520)	1.35

Day 6/7; ↑

Gene Classification Group	Score ($-log_{10}$)
**Splicesome** KEGG (cfa03040)	9.09
**Ubiquitin Mediated Proteolysis** KEGG (cfa04120)	8.39
**Proteasome** KEGG (cfa03050)	7.77
**Lysosome** KEGG (cfa04142)	6.12
**RNA Degradation** KEGG (cfa03018)	4.47
**TGF-Beta Signaling Pathway** KEGG (cfa04350)	3.34
**Cell Cycle** KEGG (cfa04110)	2.98
**Colorectal Cancer** KEGG (cfa05210)	2.58
**p53 Signaling Pathway** KEGG (cfa04115)	2.51
**Pancreatic Cancer** KEGG (cfa05212)	2.39
**Wnt Signaling Pathway** KEGG (cfa04310)	2.34
**Adherens Junction** KEGG (cfa04520)	2.30
**Aminoacyl-tRNA Biosynthesis** KEGG (cfa00970)	2.22
**Nucleotide Excision Repair** KEGG (cfa03420)	2.22
**Oocyte Meiosis** KEGG (cfa04114)	2.10
**Pathways in Cancer** KEGG (cfa05200)	2.02
**Focal Adhesion** KEGG (cfa04510)	1.76
**Toll-Like Receptor Signaling Pathway** KEGG (cfa04620)	1.76
**Basal Transcription Factors** KEGG (cfa03022)	1.49
**Pyrimidine Metabolism** KEGG (cfa00240)	1.34

Downregulated Genes

Day 3/4; ↓

Gene Classification Group	Score ($-log_{10}$)
**Phenylalanine, Tyrosine, and Tryptophan Biosynthesis** KEGG (cfa00400)	3.13
**Phenylalanine Metabolism** KEGG (cfa00360)	2.80
**Tyrosine Metabolism** KEGG (cfa00350)	1.90
**PPAR Signaling Pathway** KEGG (cfa03320)	1.88

Day 6/7; ↓

Gene Classification Group	Score ($-log_{10}$)
**Glycerolipid Metabolism** KEGG (cfa00561)	1.62
**Arachidonic Acid Metabolism** KEGG (cfa00590)	1.61
**Jak-STAT Signaling Pathway** KEGG (cfa04630)	1.51
**Complement and Coagulation Cascades** KEGG (cfa04610)	1.44
**Cytokine-Cytokine Receptor Interaction** KEGG (cfa04060)	1.39
**Arginine and Proline Metabolism** KEGG (cfa00330)	1.31
