# Supplementary material for: Influenza Transmission in the Mother-Infant Dyad Leads to Severe Disease, Mammary Gland Infection, and Pathogenesis by Regulating Host Responses
Source: PLoS Pathog. 2015 Oct 8;11(10):e1005173. doi: 10.1371/journal.ppat.1005173 (PMC4598190; doi:10.1371/journal.ppat.1005173)
Supplement: S3 Table — (PDF) [file ppat.1005173.s003.pdf]

S3 Table. qRT-PCR Primer Sequences

Ferret Gene Targets	
**Gene**	Primer Sequence (5' - 3')
**β-ACTIN**	Forward: TGACCGGATGCAGAAGGA
	Reverse: CCGATCCACACCGAGTACTT
**STAT5a**	Forward: CCCAAGGACGAGGTCTTCTC
	Reverse: CACTGCTTTAGCGAGCACAG
**STAT5b**	Forward: AGCTCCAGAACACGTACGAC
	Reverse: GTCAGCAAGACTTCCAACCG
**LPL**	Forward: AGTTCATCAACTGGATGGCG
	Reverse: GACCTTCTTATTGGTCAGGCTT
**CSN2**	Forward: CCACTGAGACTGTGGAAAGC
	Reverse: TTCTGCTTGTTGATGTGCGT
**FOS**	Forward: GGGAAAGGAATAAGATGGCCG
	Reverse: TCTGTTTCCGCTTGGAGTGT
**MSR1**	Forward: GCCTTGCTCCCTCTGTATCC
	Reverse: GTGCAGCTTTGAGGGACTTC
**CXCL10**	Forward: CTTTGAACCAAAGTGCTGTTCTTATC
	Reverse: AGCGTGTAGTTCTAGAGAGAGGTACTC
**CCL2**	Forward: GCCCAGCCAGATGCAATTA
	Reverse: TTCTTTGGGACACTTGCTGC
**CCL19**	Forward: TTCCGAGCTGGAACACTTCT
	Reverse: GAACACAACAGCAGGCACTC
**Influenza Gene Targets**	
**Gene**	Primer Sequence (5' - 3')
**A/California/07/2009 (H1N1) Segment 7**	Forward: AAGACAAGACCAATCTTGTCACCTCT
	Reverse: TCTACGCTGCAGTCCTCGCT
