# Supplementary material for: Influenza Transmission in the Mother-Infant Dyad Leads to Severe Disease, Mammary Gland Infection, and Pathogenesis by Regulating Host Responses
Source: PLoS Pathog. 2015 Oct 8;11(10):e1005173. doi: 10.1371/journal.ppat.1005173 (PMC4598190; doi:10.1371/journal.ppat.1005173)
Supplement: S4 Table — (PDF) [file ppat.1005173.s004.pdf]

S4 Table. Functional Annotation Analysis for Bystander Mammary Glands

Upregulated Genes

Day 3/4; ↑

Gene Classification Group	Score (-log10)
**Complement and Coagulation Cascades** KEGG (cfa04610)	2.03
**Small Cell Lung Cancer** KEGG (cfa05222)	1.75
**Prostate Cancer** KEGG (cfa05215)	1.65
**Focal Adhesion** KEGG (cfa04510)	1.42
**Arachidonic Acid Metabolism** KEGG (cfa00590)	1.38
**Chemokine Signaling Pathway** KEGG (cfa04062)	1.38
**Neutrophin Signaling Pathway** KEGG (cfa04722)	1.38

Day 6/7; ↑

Gene Classification Group	Score (-log10)
**Proteasome** KEGG (cfa03050)	1.90
**Ether Lipid Metabolism** KEGG (cfa00565)	1.89
**Complement and Coagulation Cascades** KEGG (cfa04610)	1.79
**Leukocyte Transendothelial Migration** KEGG (cfa04670)	1.47
**ECM-Receptor Interaction** KEGG (cfa04512)	1.40

Downregulated Genes

Day 3/4; ↓

Gene Classification Group	Score (-log10)
**Hypertrophic Cardiomyopathy (HCM)** KEGG (cfa05410)	2.03
**Dilated Cardiomyopathy** KEGG (cfa05414)	1.84
**Calcium Signaling Pathway** KEGG (cfa04020)	1.69
**Valine, Leucine, and Isoleucine Biosynthesis** KEGG (cfa00290)	1.68
**RNA Polymerase** KEGG (cfa03020)	1.35

Day 6/7; ↓

Gene Classification Group	Score (-log10)
**Glycerolipid Metabolism** KEGG (cfa00561)	2.66
**Propanoate Metabolism** KEGG (cfa00640)	2.60
**Hedgehog Signaling Pathway** KEGG (cfa04340)	1.76
**PPAR Signaling Pathway** KEGG (cfa03320)	1.76
**Adipocytokine Signaling Pathway** KEGG (cfa04920)	1.72
**Glycosphingolipid Biosynthesis** KEGG (cfa00604)	1.67
**Fructose and Mannose Metabolism** KEGG (cfa00051)	1.63
**N-Glycan Biosynthesis** KEGG (cfa00510)	1.62
**Valine, Leucine, and Isoleucine Degradation** KEGG (cfa00280)	1.62
**Acute Myeloid Leukemia** KEGG (cfa05221)	1.40
**Pentose Phosphate Pathway** KEGG (cfa00030)	1.39
**Tight Junction** KEGG (cfa04530)	1.36
