# Supplementary material for: Influenza Transmission in the Mother-Infant Dyad Leads to Severe Disease, Mammary Gland Infection, and Pathogenesis by Regulating Host Responses
Source: PLoS Pathog. 2015 Oct 8;11(10):e1005173. doi: 10.1371/journal.ppat.1005173 (PMC4598190; doi:10.1371/journal.ppat.1005173)
Supplement: S5 Table — (PDF) [file ppat.1005173.s005.pdf]

S5 Table. Functional Annotation Analysis for Bystander / H1N1+ Mammary Gland Comparison

i. KEGG Pathway Annotations

Upregulated Genes; Day 3/4

H1N1+

Gene Classification Group	Score (-log_{10})	Gene Count	Fold Enrichment
cfa04520:Adherens junction	**1.92**	10	2.64
cfa03018:RNA degradation	**1.52**	8	2.62
cfa05322:Systemic lupus erythematosus	**1.35**	10	2.11
cfa04010:MAPK signaling pathway	1.19	21	1.50
cfa03040:Spliceosome	1.17	13	1.74
cfa04120:Ubiquitin mediated proteolysis	1.15	12	1.78
cfa00480:Glutathione metabolism	1.14	6	2.65
cfa04530:Tight junction	1.13	12	1.77
cfa04622:RIG-I-like receptor signaling pathway	1.12	7	2.33
cfa04722:Neurotrophin signaling pathway	0.98	11	1.71

Bystander

Gene Classification Group	Score (-log_{10})	Gene Count	Fold Enrichment
cfa04510:Focal adhesion	**2.60**	19	2.17
cfa04664:Fc epsilon RI signaling pathway	**1.78**	9	2.71
cfa00590:Arachidonic acid metabolism	1.20	6	2.77
cfa04910:Insulin signaling pathway	1.20	11	1.89
cfa04010:MAPK signaling pathway	1.20	19	1.54
cfa04610:Complement and coagulation cascades	1.20	7	2.45
cfa05222:Small cell lung cancer	1.19	8	2.23
cfa04810:Regulation of actin cytoskeleton	1.12	15	1.62
cfa04062:Chemokine signaling pathway	1.08	13	1.68
cfa05221:Acute myeloid leukemia	0.97	6	2.37

Both

Gene Classification Group	Score (-log_{10})	Gene Count	Fold Enrichment
cfa03040:Spliceosome	**3.95**	13	3.84
cfa04612:Antigen processing and presentation	**2.09**	7	3.91
cfa05200:Pathways in cancer	**1.42**	14	1.83
cfa04310:Wnt signaling pathway	**1.37**	8	2.45
cfa02010:ABC transporters	1.14	4	4.09
cfa04120:Ubiquitin mediated proteolysis	1.09	7	2.29
cfa04620:Toll-like receptor signaling pathway	0.84	5	2.44
cfa04722:Neurotrophin signaling pathway	0.79	6	2.06
cfa05215:Prostate cancer	0.78	5	2.30
cfa04610:Complement and coagulation cascades	0.74	4	2.70

Upregulated Genes; Day 6/7

H1N1+

Gene Classification Group	Score ($-\log_{10}$)	Gene Count	Fold Enrichment
cfa03040:Spliceosome	**8.91**	52	2.35
cfa04120:Ubiquitin mediated proteolysis	**7.59**	46	2.31
cfa04142:Lysosome	**4.58**	35	2.06
cfa03050:Proteasome	**4.06**	19	2.65
cfa03018:RNA degradation	**4.06**	22	2.44
cfa04110:Cell cycle	**3.57**	35	1.86
cfa04350:TGF-beta signaling pathway	**3.21**	25	2.03
cfa04310:Wnt signaling pathway	**2.54**	35	1.64
cfa00970:Aminoacyl-tRNA biosynthesis	**2.49**	15	2.29
cfa04520:Adherens junction	**2.26**	21	1.87

Bystander

Gene Classification Group	Score ($-\log_{10}$)	Gene Count	Fold Enrichment
cfa04740:Olfactory transduction	**3.81**	27	2.19
cfa03010:Ribosome	**1.92**	9	2.87
cfa00591:Linoleic acid metabolism	0.90	3	4.83
cfa03320:PPAR signaling pathway	0.86	5	2.49
cfa04080:Neuroactive ligand-receptor interaction	0.85	11	1.60
cfa00072:Synthesis and degradation of ketone bodies	0.72	2	9.67
cfa00512:O-Glycan biosynthesis	0.70	3	3.62
cfa04270:Vascular smooth muscle contraction	0.65	6	1.83
cfa04960:Aldosterone-regulated sodium reabsorption	0.48	3	2.54
cfa00270:Cysteine and methionine metabolism	0.48	3	2.54

Both

Gene Classification Group	Score ($-\log_{10}$)	Gene Count	Fold Enrichment
cfa03050:Proteasome	**2.44**	10	3.14
cfa04610:Complement and coagulation cascades	**2.03**	11	2.56
cfa04612:Antigen processing and presentation	**1.88**	12	2.31
cfa00565:Ether lipid metabolism	**1.63**	7	3.06
cfa04620:Toll-like receptor signaling pathway	**1.48**	12	2.01
cfa04512:ECM-receptor interaction	**1.39**	11	2.03
cfa05222:Small cell lung cancer	**1.39**	11	2.03
cfa04670:Leukocyte transendothelial migration	1.26	14	1.74
cfa04510:Focal adhesion	1.19	20	1.52
cfa00520:Amino sugar and nucleotide sugar metabolism	1.18	7	2.40

Downregulated Genes; Day 3/4

H1N1+

Gene Classification Group	Score (-$\log_{10}$)	Gene Count	Fold Enrichment
cfa00400:Phenylalanine, tyrosine and tryptophan biosynthesis	**2.20**	4	9.21
cfa00360:Phenylalanine metabolism	**1.62**	5	4.32
cfa04340:Hedgehog signaling pathway	**1.60**	9	2.49
cfa00590:Arachidonic acid metabolism	**1.31**	8	2.35
cfa03320:PPAR signaling pathway	1.27	10	2.03
cfa00270:Cysteine and methionine metabolism	1.19	7	2.42
cfa00130:Ubiquinone and other terpenoid-quinone biosynthesis	1.07	3	5.92
cfa04080:Neuroactive ligand-receptor interaction	0.96	23	1.37
cfa00430:Taurine and hypotaurine metabolism	0.87	3	4.61
cfa00620:Pyruvate metabolism	0.83	6	2.13

Bystander

Gene Classification Group	Score (-$\log_{10}$)	Gene Count	Fold Enrichment
cfa05410:Hypertrophic cardiomyopathy (HCM)	**1.53**	5	4.20
cfa05414:Dilated cardiomyopathy	**1.45**	5	3.95
cfa00563:GPI-anchor biosynthesis	**1.38**	3	9.04
cfa00350:Tyrosine metabolism	1.14	3	6.63
cfa00980:Metabolism of xenobiotics by cytochrome P450	1.00	3	5.52
cfa04020:Calcium signaling pathway	0.95	6	2.31
cfa00982:Drug metabolism	0.91	3	4.85
cfa00360:Phenylalanine metabolism	0.67	2	8.29
cfa05214:Glioma	0.62	3	3.16
cfa04260:Cardiac muscle contraction	0.61	3	3.11

Both

Gene Classification Group	Score (-$\log_{10}$)	Gene Count	Fold Enrichment
cfa00290:Valine, leucine and isoleucine biosynthesis	**2.00**	4	8.38
cfa05213:Endometrial cancer	**1.49**	7	2.88
cfa00970:Aminoacyl-tRNA biosynthesis	**1.33**	6	2.99
cfa04012:ErbB signaling pathway	0.99	8	2.00
cfa00030:Pentose phosphate pathway	0.99	4	3.49
cfa05223:Non-small cell lung cancer	0.98	6	2.37
cfa04020:Calcium signaling pathway	0.94	13	1.58
cfa04510:Focal adhesion	0.93	14	1.54
cfa05212:Pancreatic cancer	0.92	7	2.07
cfa05410:Hypertrophic cardiomyopathy (HCM)	0.77	7	1.86

Downregulated Genes; Day 6/7

H1N1+

Gene Classification Group	Score (-log$_{10}$)	Gene Count	Fold Enrichment
cfa04060:Cytokine-cytokine receptor interaction	**1.87**	20	1.81
cfa00590:Arachidonic acid metabolism	1.22	7	2.48
cfa04740:Olfactory transduction	1.08	33	1.32
cfa04630:Jak-STAT signaling pathway	0.89	13	1.56
cfa05414:Dilated cardiomyopathy	0.89	9	1.78
cfa03020:RNA polymerase	0.85	4	3.02
cfa00230:Purine metabolism	0.82	13	1.50
cfa04610:Complement and coagulation cascades	0.78	7	1.88
cfa00650:Butanoate metabolism	0.63	4	2.38
cfa04914:Progesterone-mediated oocyte maturation	0.53	8	1.48

Bystander

Gene Classification Group	Score (-log$_{10}$)	Gene Count	Fold Enrichment
cfa00640:Propanoate metabolism	**2.45**	10	3.05
cfa00510:N-Glycan biosynthesis	**1.41**	10	2.13
cfa04340:Hedgehog signaling pathway	**1.39**	11	2.01
cfa03060:Protein export	**1.32**	4	4.58
cfa00604:Glycosphingolipid biosynthesis	1.24	5	3.27
cfa04950:Maturity onset diabetes of the young	1.08	6	2.50
cfa04916:Melanogenesis	1.02	16	1.53
cfa04720:Long-term potentiation	1.00	12	1.66
cfa00250:Alanine, aspartate and glutamate metabolism	1.00	7	2.14
cfa00830:Retinol metabolism	0.95	8	1.93

Both

Gene Classification Group	Score (-log$_{10}$)	Gene Count	Fold Enrichment
cfa05221:Acute myeloid leukemia	**2.16**	10	2.87
cfa00561:Glycerolipid metabolism	**1.88**	8	3.08
cfa00330:Arginine and proline metabolism	**1.82**	9	2.73
cfa03320:PPAR signaling pathway	1.20	9	2.09
cfa04920:Adipocytokine signaling pathway	0.97	8	1.97
cfa04530:Tight junction	0.95	13	1.59
cfa00350:Tyrosine metabolism	0.93	5	2.63
cfa00051:Fructose and mannose metabolism	0.93	5	2.63
cfa00520:Amino sugar and nucleotide sugar metabolism	0.91	6	2.25
cfa04810:Regulation of actin cytoskeleton	0.88	18	1.41

S5 Table. Functional Annotation Analysis for Bystander / H1N1+ Mammary Gland Comparison

ii. Gene Ontology – Biological Process Annotations

Upregulated Genes; Day 3/4

H1N1+

Gene Classification Group	Score (-log$_{10}$)	Gene Count	Fold Enrichment
GO:0050790~regulation of catalytic activity	**4.02**	10	4.69
GO:0065009~regulation of molecular function	**3.58**	10	4.15
GO:0051336~regulation of hydrolase activity	**2.43**	6	5.22
GO:0043085~positive regulation of catalytic activity	**2.25**	6	4.77
GO:0044093~positive regulation of molecular function	**2.01**	6	4.22
GO:0012502~induction of programmed cell death	**1.65**	4	6.09
GO:0006917~induction of apoptosis	**1.65**	4	6.09
GO:0048070~regulation of pigmentation during development	**1.60**	3	10.97
GO:0048518~positive regulation of biological process	**1.51**	11	2.01
GO:0043933~macromolecular complex subunit organization	**1.47**	4	5.22

Bystander

Gene Classification Group	Score (-log$_{10}$)	Gene Count	Fold Enrichment
GO:0065008~regulation of biological quality	**1.94**	14	2.02
GO:0065007~biological regulation	**1.62**	31	1.33
GO:0043687~post-translational protein modification	**1.40**	7	2.62
GO:0043412~biopolymer modification	1.29	8	2.25
GO:0006464~protein modification process	1.29	8	2.25
GO:0042311~vasodilation	1.18	3	6.74
GO:0019725~cellular homeostasis	1.18	6	2.62
GO:0000041~transition metal ion transport	1.08	3	5.90
GO:0044267~cellular protein metabolic process	1.02	9	1.84
GO:0015674~di-, tri-valent inorganic cation transport	0.98	3	5.24

Both

Gene Classification Group	Score (-log$_{10}$)	Gene Count	Fold Enrichment
GO:0044260~cellular macromolecule metabolic process	1.00	8	1.86
GO:0034641~cellular nitrogen compound metabolic process	0.95	6	2.18
GO:0043170~macromolecule metabolic process	0.93	9	1.67
GO:0006807~nitrogen compound metabolic process	0.87	6	2.07
GO:0008380~RNA splicing	0.86	2	13.10
GO:0006520~cellular amino acid metabolic process	0.73	2	9.36
GO:0006396~RNA processing	0.67	2	8.19
GO:0006397~mRNA processing	0.67	2	8.19
GO:0016071~mRNA metabolic process	0.67	2	8.19
GO:0010467~gene expression	0.64	4	2.30

Upregulated Genes; Day 6/7

H1N1+

Gene Classification Group	Score (-log$_{10}$)	Gene Count	Fold Enrichment
GO:0043085~positive regulation of catalytic activity	**3.35**	10	3.80
GO:0050790~regulation of catalytic activity	**3.20**	13	2.91
GO:0009987~cellular process	**3.17**	61	1.33
GO:0044093~positive regulation of molecular function	**2.90**	10	3.36
GO:0048518~positive regulation of biological process	**2.72**	22	1.92
GO:0065009~regulation of molecular function	**2.68**	13	2.58
GO:0048522~positive regulation of cellular process	**2.52**	20	1.94
GO:0007242~intracellular signaling cascade	**2.34**	14	2.26
GO:0006470~protein amino acid dephosphorylation	**2.29**	4	8.73
GO:0016311~dephosphorylation	**2.29**	4	8.73

Bystander

Gene Classification Group	Score (-log$_{10}$)	Gene Count	Fold Enrichment
GO:0042180~cellular ketone metabolic process	1.24	4	4.28
GO:0019752~carboxylic acid metabolic process	1.24	4	4.28
GO:0043436~oxoacid metabolic process	1.24	4	4.28
GO:0006082~organic acid metabolic process	1.24	4	4.28
GO:0016043~cellular component organization	1.11	7	2.21
GO:0044271~nitrogen compound biosynthetic process	1.09	4	3.74
GO:0034641~cellular nitrogen compound metabolic process	1.08	8	2.00
GO:0006807~nitrogen compound metabolic process	0.98	8	1.89
GO:0043933~macromolecular complex subunit organization	0.92	3	4.81
GO:0019217~regulation of fatty acid metabolic process	0.91	2	14.97

Both

Gene Classification Group	Score (-log$_{10}$)	Gene Count	Fold Enrichment
GO:0044238~primary metabolic process	**2.44**	32	1.53
GO:0006807~nitrogen compound metabolic process	**2.03**	16	1.95
GO:0044237~cellular metabolic process	**1.89**	28	1.49
GO:0034641~cellular nitrogen compound metabolic process	**1.85**	15	1.93
GO:0044260~cellular macromolecule metabolic process	**1.72**	20	1.64
GO:0006826~iron ion transport	**1.69**	3	11.56
GO:0000041~transition metal ion transport	**1.61**	4	5.78
GO:0008152~metabolic process	**1.58**	36	1.31
GO:0015674~di-, tri-valent inorganic cation transport	**1.47**	4	5.14
GO:0010467~gene expression	**1.35**	10	2.03

Downregulated Genes; Day 3/4

H1N1+

Gene Classification Group	Score (-log$_{10}$)	Gene Count	Fold Enrichment
GO:0055114~oxidation reduction	**1.96**	12	2.25
GO:0002376~immune system process	**1.80**	16	1.85
GO:0006955~immune response	1.20	12	1.75
GO:0006357~regulation of transcription from RNA polymerase (…)	1.03	5	2.77
GO:0022900~electron transport chain	0.99	4	3.41
GO:0009887~organ morphogenesis	0.97	6	2.29
GO:0007423~sensory organ development	0.97	5	2.64
GO:0051924~regulation of calcium ion transport	0.91	3	4.74
GO:0045449~regulation of transcription	0.81	9	1.66
GO:0010959~regulation of metal ion transport	0.81	3	4.15

Bystander

Gene Classification Group	Score (-log$_{10}$)	Gene Count	Fold Enrichment
GO:0032501~multicellular organismal process	**1.72**	20	1.57
GO:0006518~peptide metabolic process	**1.62**	3	11.23
GO:0006928~cell motion	**1.50**	5	3.90
GO:0009888~tissue development	**1.33**	6	2.88
GO:0048871~multicellular organismal homeostasis	1.22	3	7.02
GO:0007275~multicellular organismal development	1.20	13	1.66
GO:0016043~cellular component organization	1.17	8	2.11
GO:0048856~anatomical structure development	1.12	12	1.66
GO:0007169~transmembrane receptor protein tyrosine kinase (…)	1.12	3	6.24
GO:0035239~tube morphogenesis	1.04	3	5.61

Both

Gene Classification Group	Score (-log$_{10}$)	Gene Count	Fold Enrichment
GO:0007166~cell surface receptor linked signal transduction	0.53	4	1.93
GO:0055114~oxidation reduction	0.24	2	2.22
GO:0019219~regulation of nucleobase, nucleoside, nucleotide (…)	0.18	2	1.82
GO:0051171~regulation of nitrogen compound metabolic process	0.18	2	1.79
GO:0031326~regulation of cellular biosynthetic process	0.15	2	1.60
GO:0030154~cell differentiation	0.15	2	1.58
GO:0008152~metabolic process	0.15	5	1.03
GO:0009889~regulation of biosynthetic process	0.15	2	1.56
GO:0048869~cellular developmental process	0.14	2	1.52
GO:0043170~macromolecule metabolic process	0.13	3	1.12

Downregulated Genes; Day 6/7

H1N1+

Gene Classification Group	Score (-log$_{10}$)	Gene Count	Fold Enrichment
GO:0001568~blood vessel development	**1.31**	6	2.85
GO:0032501~multicellular organismal process	1.27	28	1.34
GO:0001944~vasculature development	1.25	6	2.73
GO:0032844~regulation of homeostatic process	1.21	3	6.83
GO:0048731~system development	1.16	17	1.51
GO:0042325~regulation of phosphorylation	1.13	5	3.00
GO:0002696~positive regulation of leukocyte activation	1.11	4	3.80
GO:0031399~regulation of protein modification process	1.11	4	3.80
GO:0001932~regulation of protein amino acid phosphorylation	1.11	4	3.80
GO:0050867~positive regulation of cell activation	1.11	4	3.80

Bystander

Gene Classification Group	Score (-log$_{10}$)	Gene Count	Fold Enrichment
GO:0043010~camera-type eye development	1.22	5	3.15
GO:0046365~monosaccharide catabolic process	1.13	3	6.14
GO:0006007~glucose catabolic process	1.13	3	6.14
GO:0006096~glycolysis	1.13	3	6.14
GO:0019320~hexose catabolic process	1.13	3	6.14
GO:0000902~cell morphogenesis	1.11	5	2.92
GO:0033036~macromolecule localization	1.04	10	1.78
GO:0007423~sensory organ development	1.01	6	2.34
GO:0044275~cellular carbohydrate catabolic process	0.95	3	4.91
GO:0016052~carbohydrate catabolic process	0.95	3	4.91

Both

Gene Classification Group	Score (-log$_{10}$)	Gene Count	Fold Enrichment
GO:0046942~carboxylic acid transport	**1.70**	4	6.24
GO:0015849~organic acid transport	**1.70**	4	6.24
GO:0008643~carbohydrate transport	**1.56**	4	5.61
GO:0015837~amine transport	1.09	3	6.02
GO:0006865~amino acid transport	1.09	3	6.02
GO:0006810~transport	0.83	17	1.35
GO:0051234~establishment of localization	0.80	17	1.33
GO:0007167~enzyme linked receptor protein signaling pathway	0.64	3	3.24
GO:0010556~regulation of macromolecule biosynthetic process	0.64	8	1.56
GO:0045449~regulation of transcription	0.63	7	1.64
