# Supplementary material for: Influenza Transmission in the Mother-Infant Dyad Leads to Severe Disease, Mammary Gland Infection, and Pathogenesis by Regulating Host Responses
Source: PLoS Pathog. 2015 Oct 8;11(10):e1005173. doi: 10.1371/journal.ppat.1005173 (PMC4598190; doi:10.1371/journal.ppat.1005173)
Supplement: S1 Fig — Nasal washes from infant ferrets and their feeding mothers were collected on Day 3, 7 and 10 post-infant inoculation (A) or post-mother inoculation (B) with 2009 H1N1 A/Cal (105 EID50). Live viral loads were assessed by titration on MDCK cells for active viral shedding throughout the time course. Adult ferrets were pair-housed and one ferret was intranasally inoculated with 2009 H1N1 A/Cal (105 EID50) (C). Viral loads were determined for collected NW from both inoculated and naïve cage mate ferrets. (PDF) [file ppat.1005173.s006.pdf]

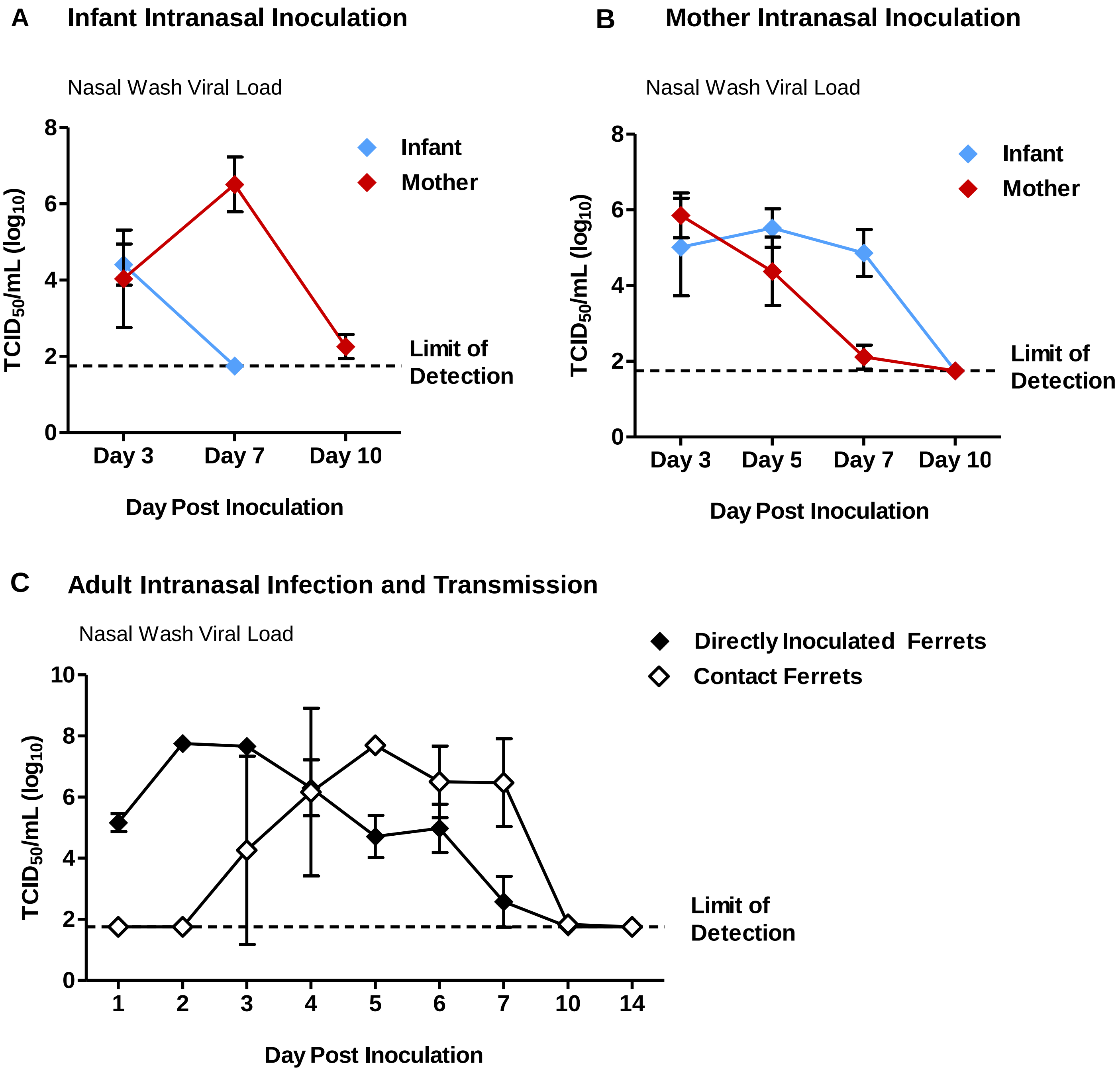
A
Infant Intranasal Inoculation
Nasal Wash Viral Load
TCID50/mL (log10)
8
6
4
2
0
Infant
Mother
Limit of Detection
Day 3
Day 7
Day 10
Day Post Inoculation
B
Mother Intranasal Inoculation
Nasal Wash Viral Load
TCID50/mL (log10)
8
6
4
2
0
Infant
Mother
Limit of Detection
Day 3
Day 5
Day 7
Day 10
Day Post Inoculation
C
Adult Intranasal Infection and Transmission
Nasal Wash Viral Load
Directly Inoculated Ferrets
Contact Ferrets
TCID50/mL (log10)
10
8
6
4
2
0
Limit of Detection
1
2
3
4
5
6
7
10
14
Day Post Inoculation
