# Supplementary material for: Influenza Transmission in the Mother-Infant Dyad Leads to Severe Disease, Mammary Gland Infection, and Pathogenesis by Regulating Host Responses
Source: PLoS Pathog. 2015 Oct 8;11(10):e1005173. doi: 10.1371/journal.ppat.1005173 (PMC4598190; doi:10.1371/journal.ppat.1005173)
Supplement: S2 Fig — (A) Clustergram of differentially regulated genes involved in Jak-STAT signaling. One-way hierarchical clustering analysis (Pearson’s correlation) was performed on all probes of significantly differentially regulated genes (≥ |1.5| fold-change in expression, p-value < 0.05) classified as members of Kyoto Encyclopedia of Genes and Genomes (KEGG) Pathway database cfa04630 (left panels). Downregulated genes are in blue, upregulated genes in red. Visualization of Jak-STAT signaling pathway by Ingenuity Pathway Analysis (IPA) (right panels). Gene expression values from D6/7 mammary glands (average of n = 3) were overlaid on IPA pathway entry Jak/STAT Signaling (blue: downregulated genes; red: upregulated genes). (B) Clustergram of differentially regulated genes involved in TGF-β signaling. One-way hierarchical clustering analysis (Pearson’s correlation) was performed on all probes of significantly differentially regulated genes (≥ |1.5| fold-change in expression, p-value < 0.05) classified as members of Kyoto Encyclopedia of Genes and Genomes (KEGG) Pathway database (left panels) cfa04350. Downregulated genes are in blue, upregulated genes in red. Visualization of TGF-β signaling pathway by Ingenuity Pathway Analysis (IPA) (right panels). Gene expression values from D6/7 mammary glands (average of n = 3) were overlaid on IPA pathway entry TGF-β Signaling (blue: downregulated genes; red: upregulated genes). (C) Clustergram of differentially regulated genes involved in Wnt-β-Catenin signaling. One-way hierarchical clustering analysis (Pearson’s correlation) was performed on all probes of significantly differentially regulated genes (≥ |1.5| fold-change in expression, p-value < 0.05) classified as members of Kyoto Encyclopedia of Genes and Genomes (KEGG) Pathway database (left panels) cfa04310. Downregulated genes are in blue, upregulated genes in red. Visualization of Wnt signaling pathway by Ingenuity Pathway Analysis (IPA) (right panels). Gene expression values from D6/7 [file ppat.1005173.s007.pdf]

Jak-STAT Signaling Pathway; cfa04630
Mammary Gland (MG)

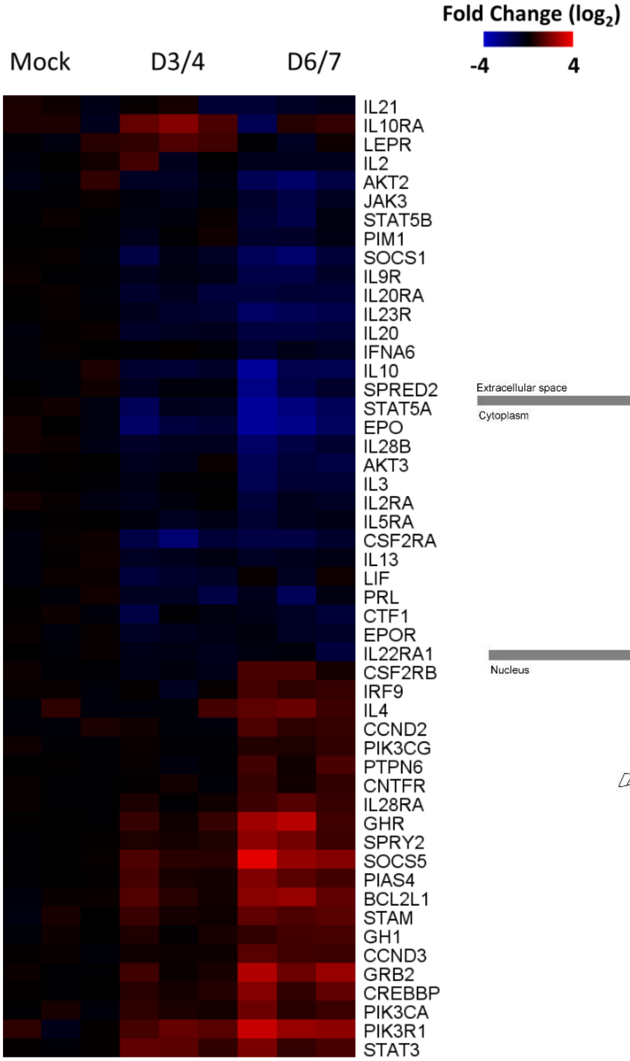

Jak/STAT Signaling
Mammary Gland (MG) D6/7

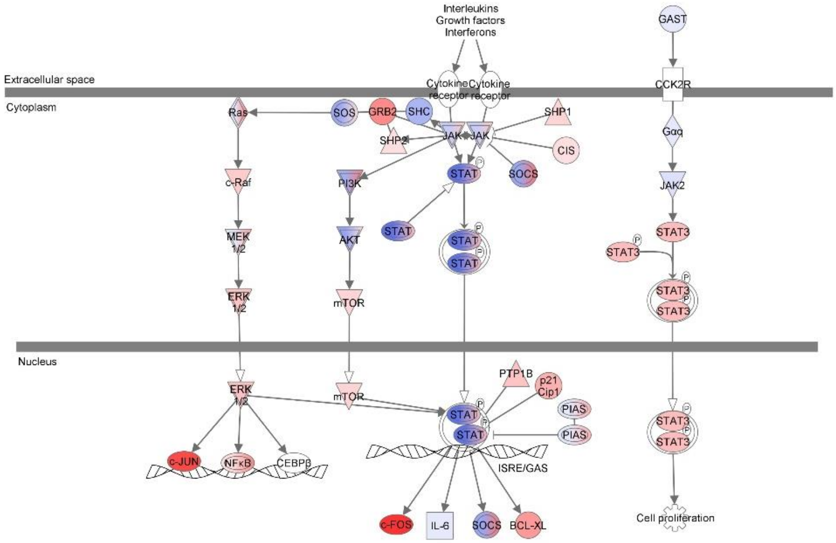

TGF-β Signaling Pathway; cfa04350
Mammary Gland (MG)

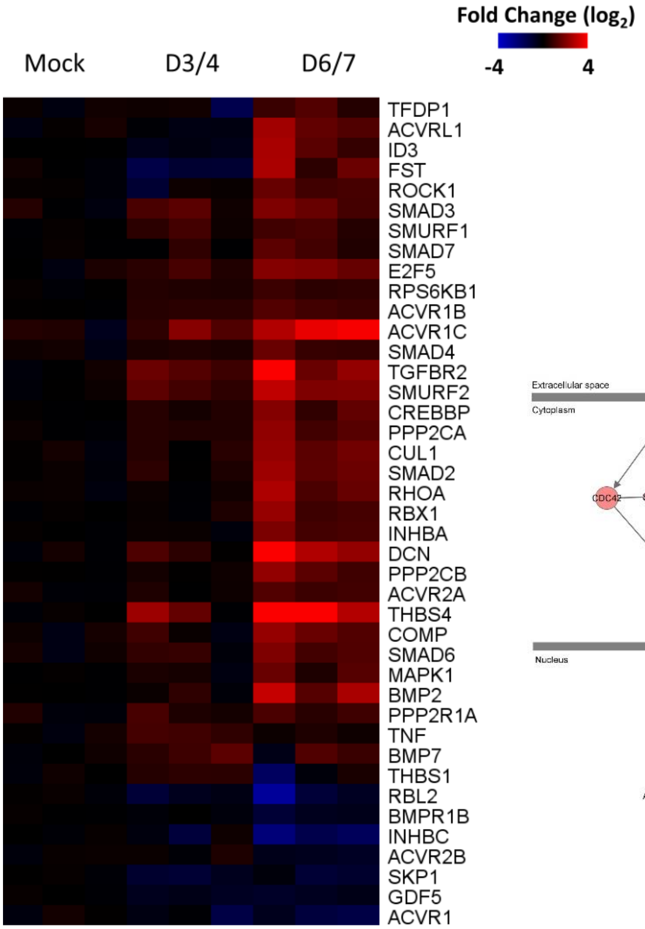

TGF-β Signaling
Mammary Gland (MG) D6/7

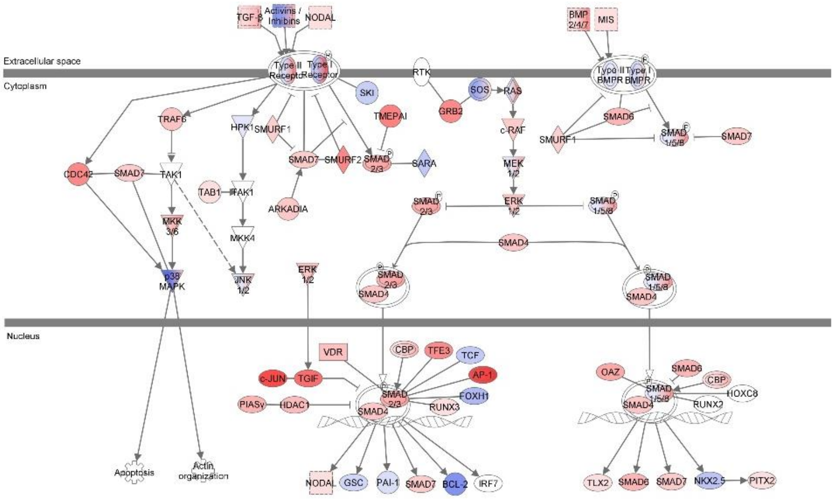

Wnt Signaling Pathway; cfa04310
Mammary Gland (MG)

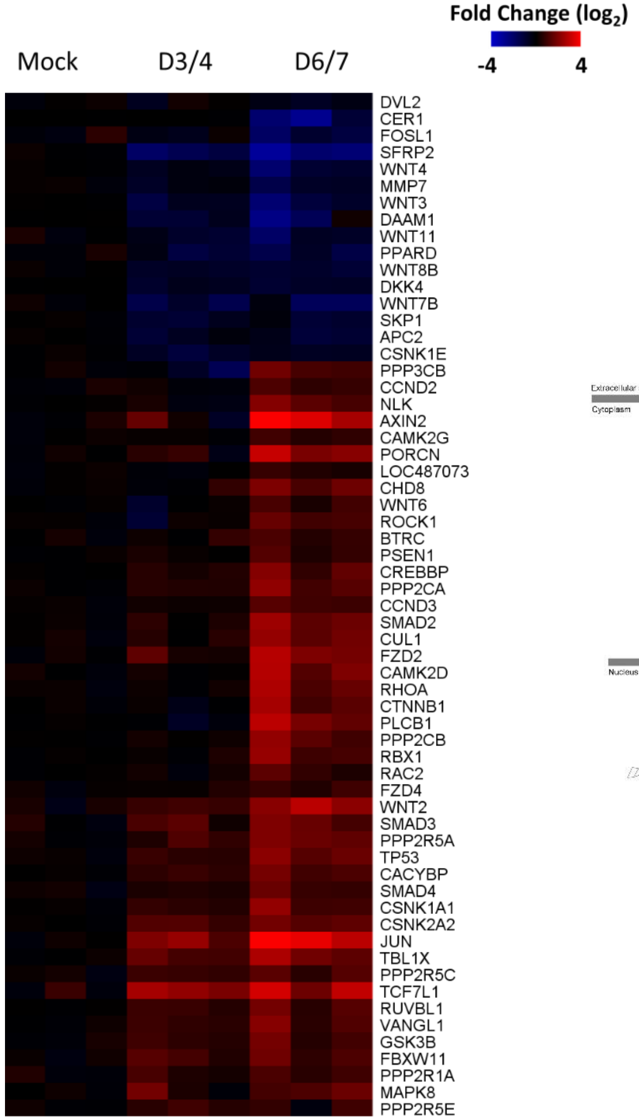

Wnt/β-Catenin Signaling
Mammary Gland (MG) D6/7

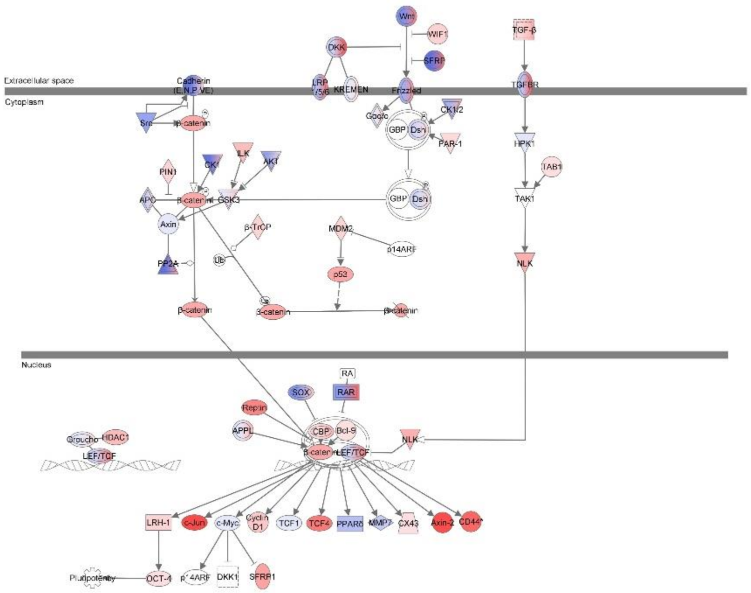

p53 Signaling Pathway; cfa04115
Mammary Gland (MG)

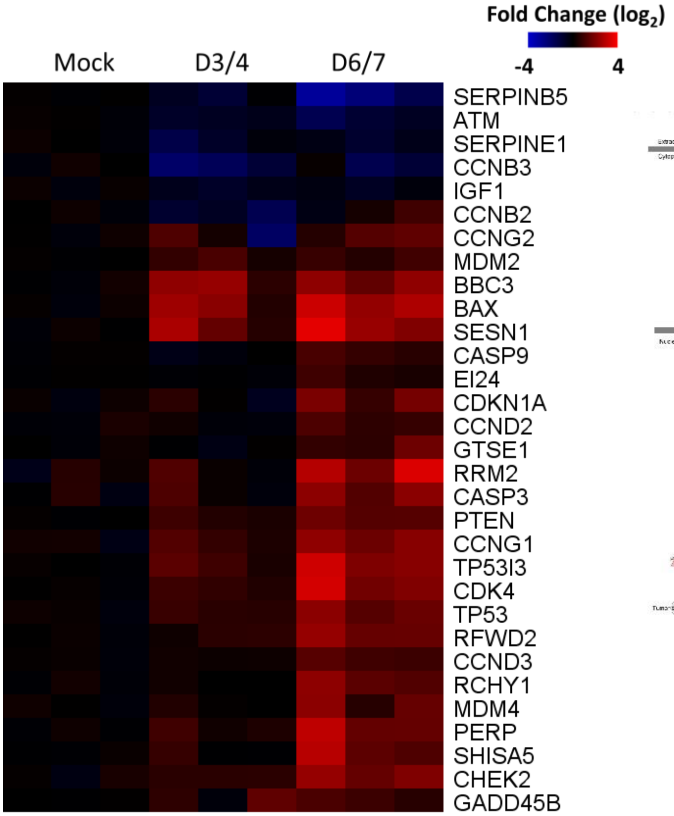

p53 Signaling
Mammary Gland (MG) D6/7

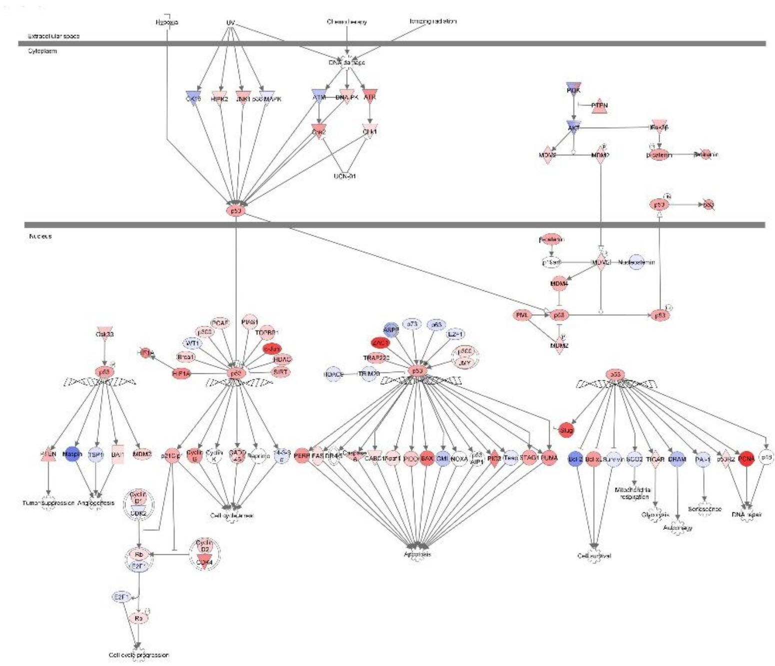

Neurotrophin Signaling Pathway; cfa04722

Mammary Gland (MG)

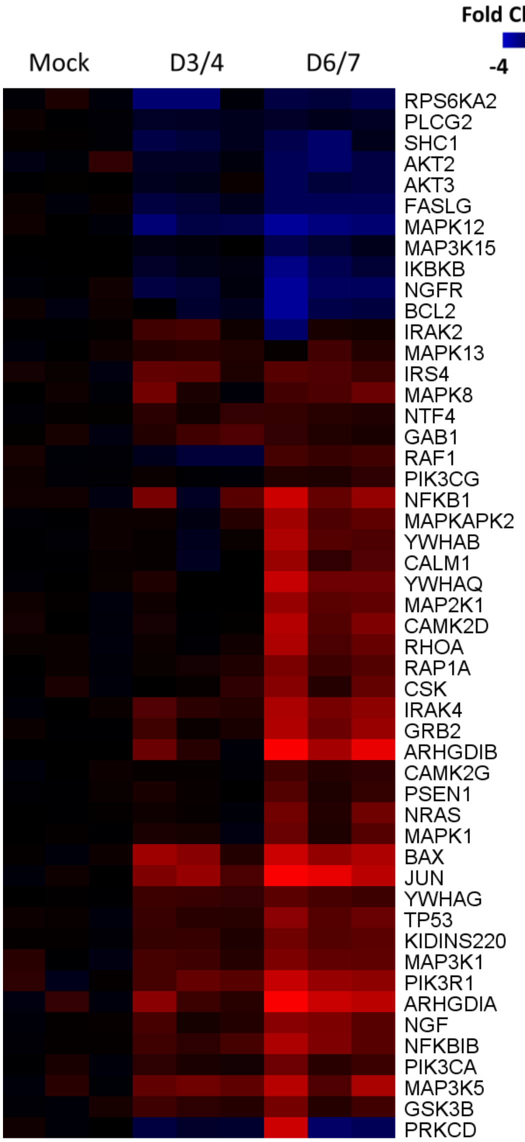

Neurotrophin/RTK Signaling

Mammary Gland (MG) D6/7

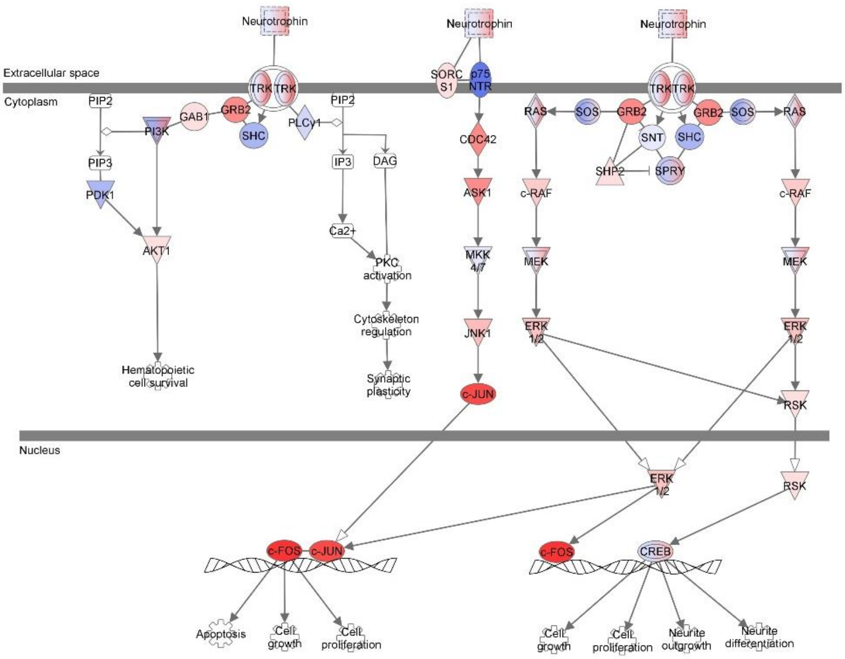

PPAR Signaling Pathway; cfa03320
Mammary Gland (MG)

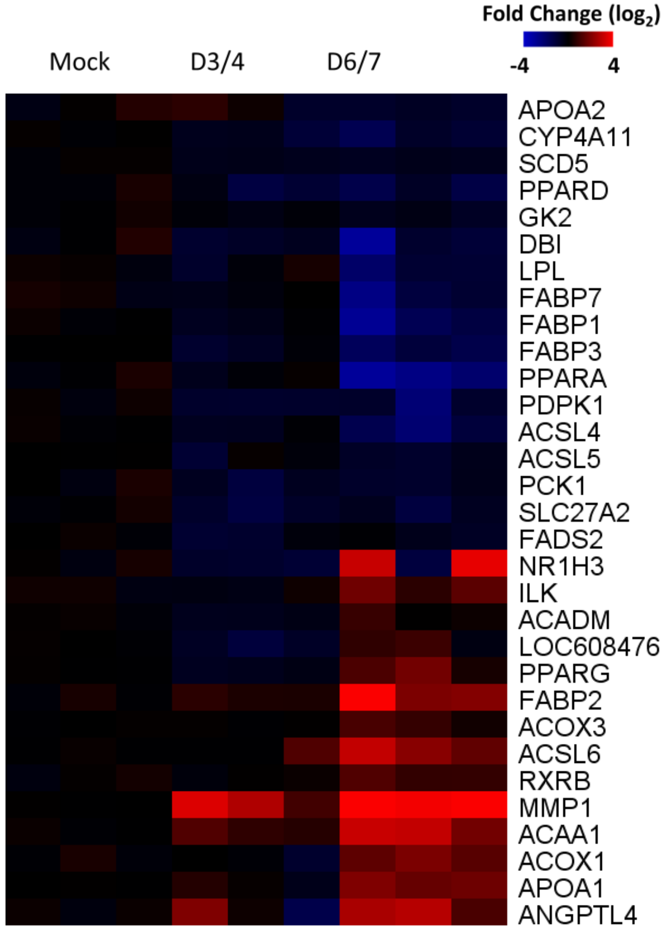

PPAR Signaling
Mammary Gland (MG) D6/7

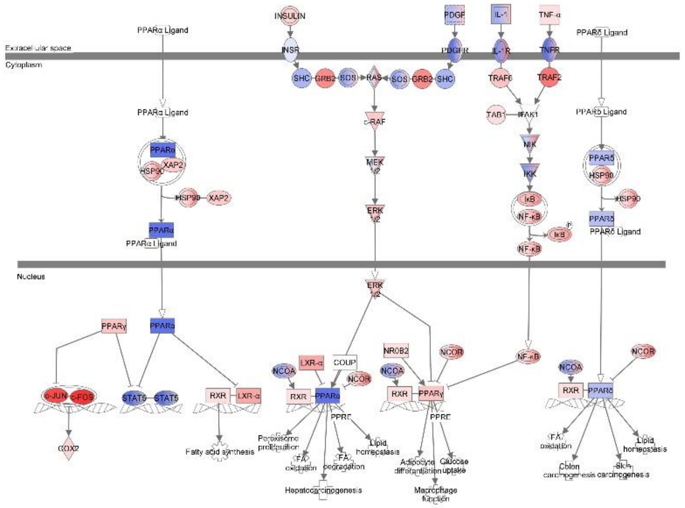

Toll-Like Receptor Signaling Pathway; cfa04620
Mammary Gland (MG)

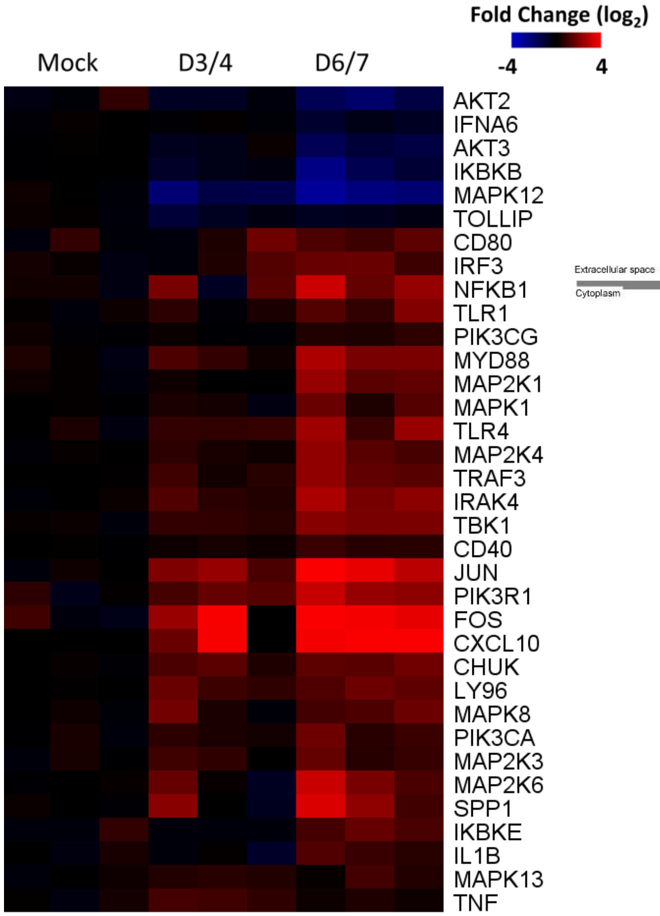

Toll-Like Receptor Signaling
Mammary Gland (MG) D6/7

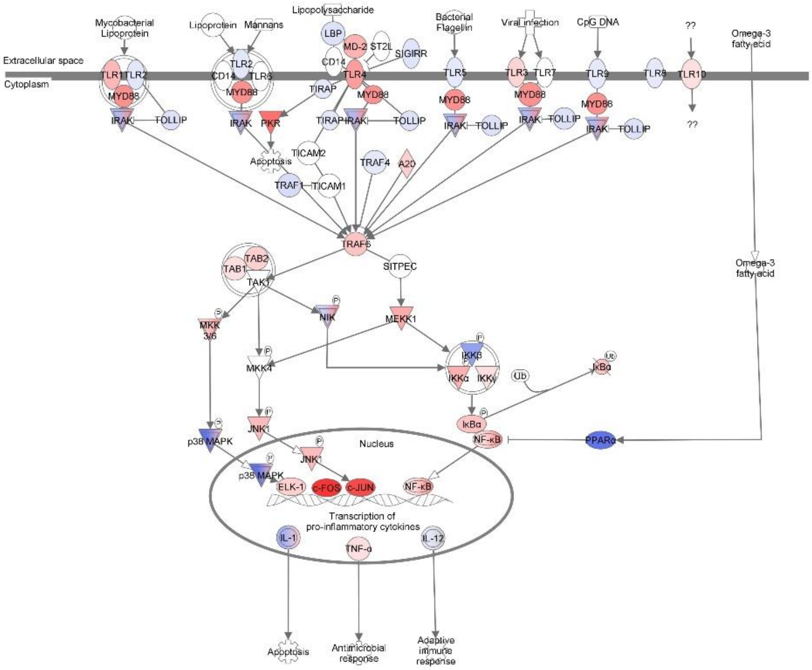

Pathways in Cancer; cfa05200
Mammary Gland (MG)

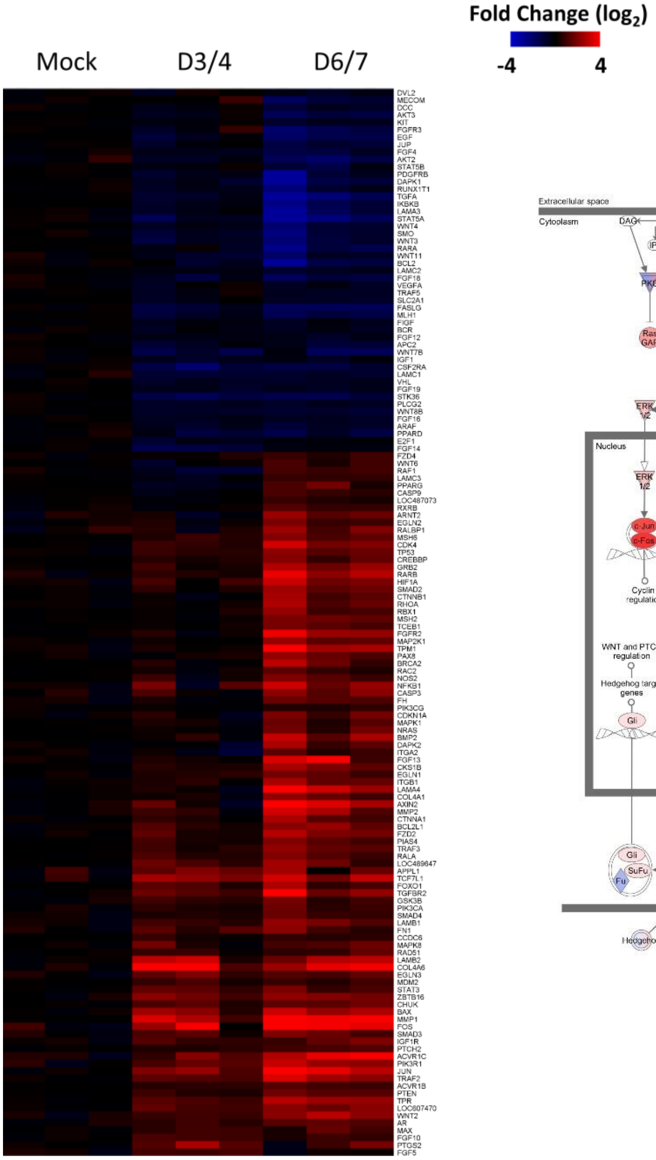

Molecular Mechanisms of Cancer
Mammary Gland (MG) D6/7

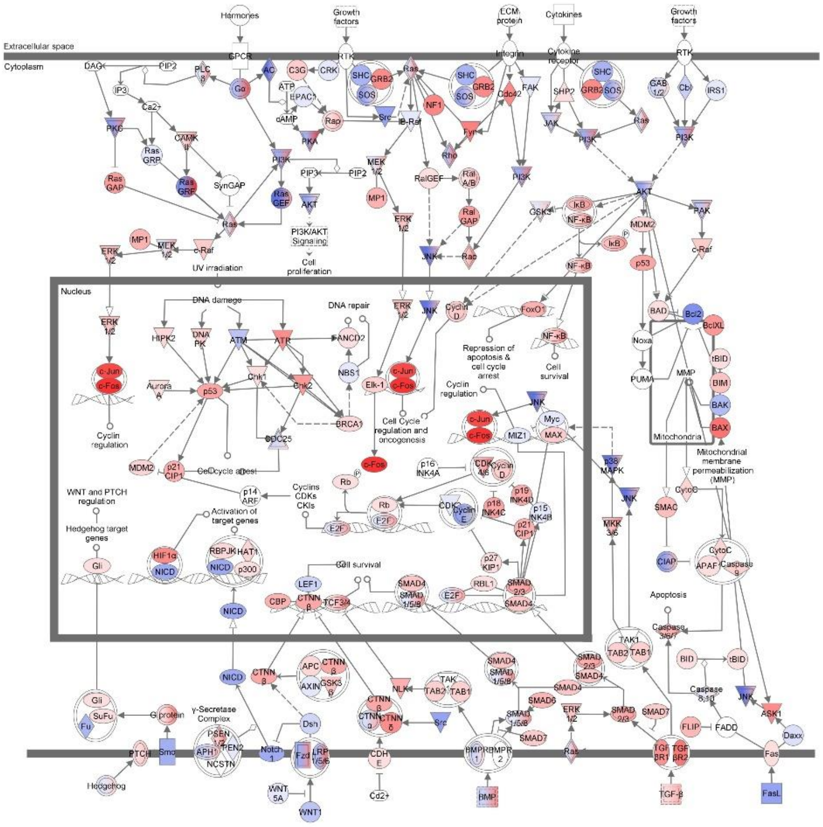

Cell Cycle; cfa04110

Mammary Gland (MG)

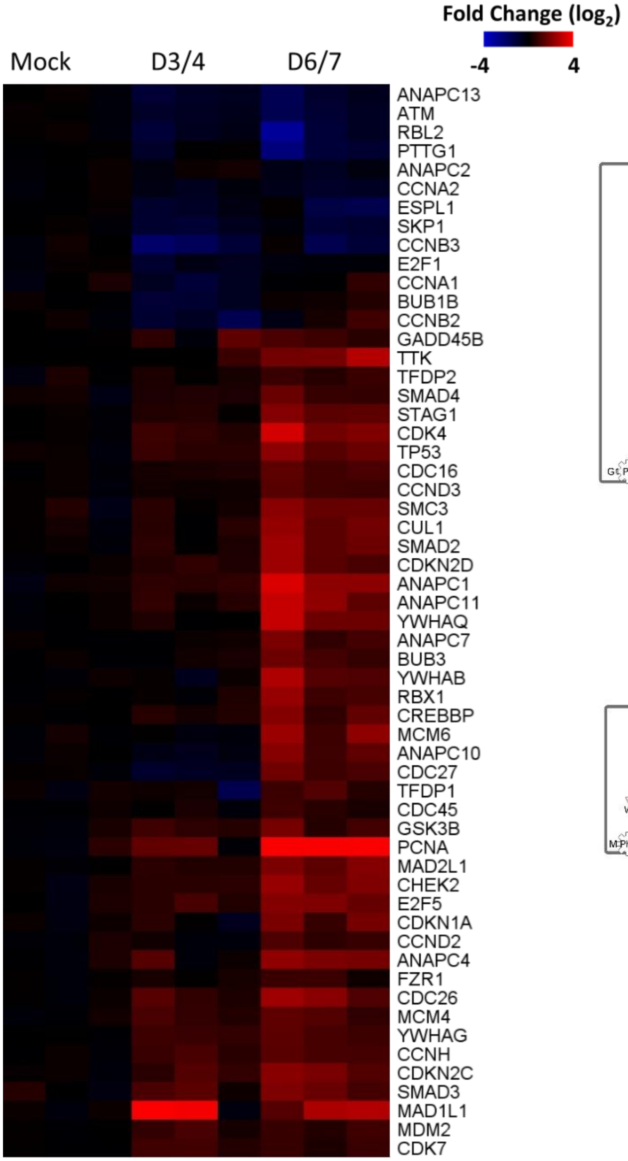

Cyclins and Cell Cycle Regulation

Mammary Gland (MG) D6/7

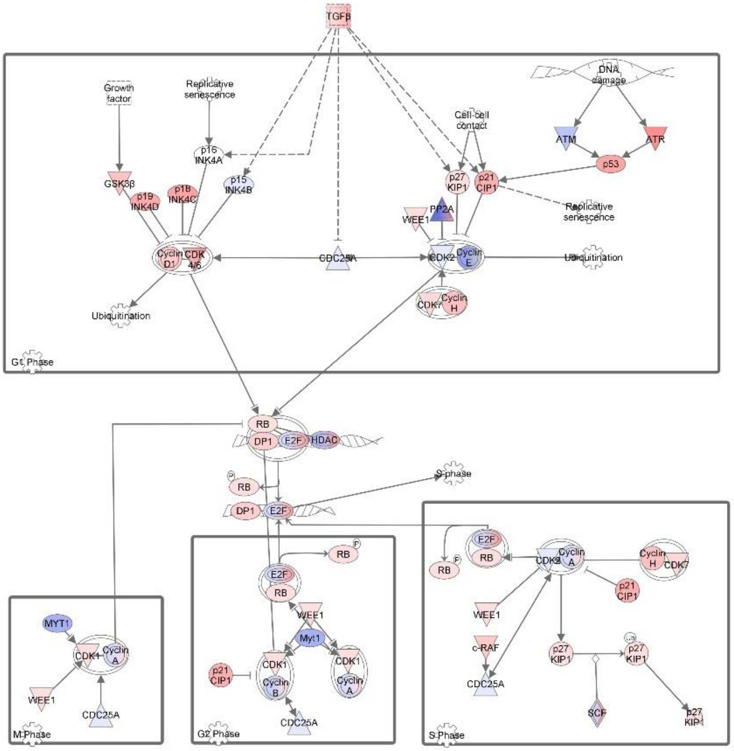

Focal Adhesion; cfa04510
Mammary Gland (MG)

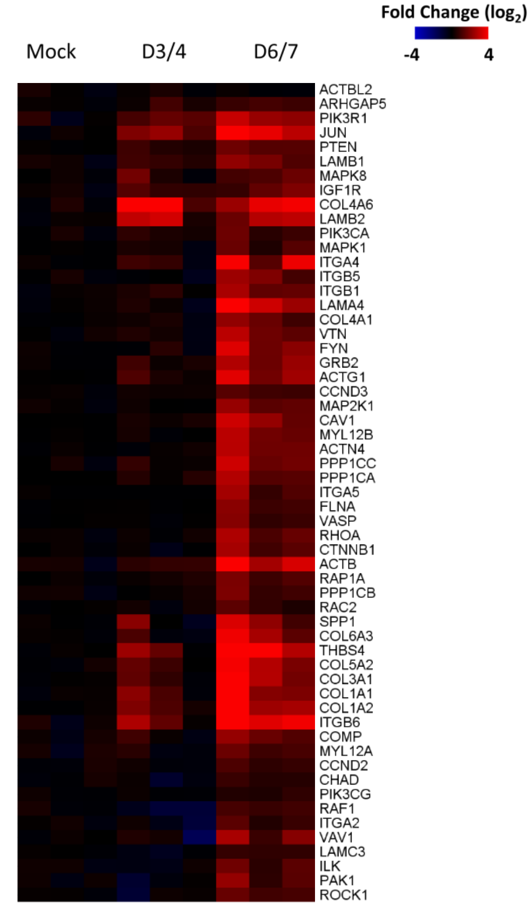

Adherens Junction; cfa04520
Mammary Gland (MG)

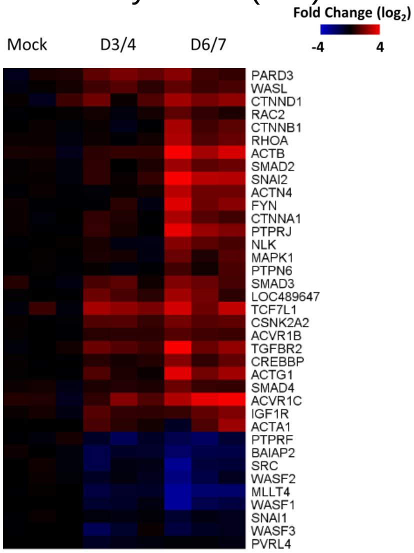

Epithelial Adherens Junction Signaling
Mammary Gland (MG) D6/7

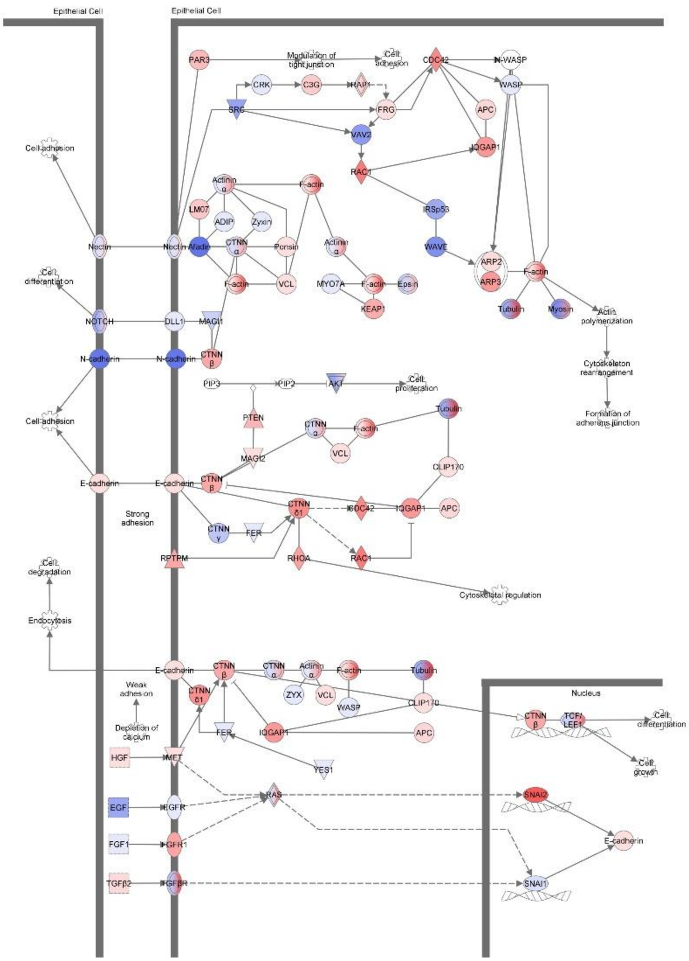

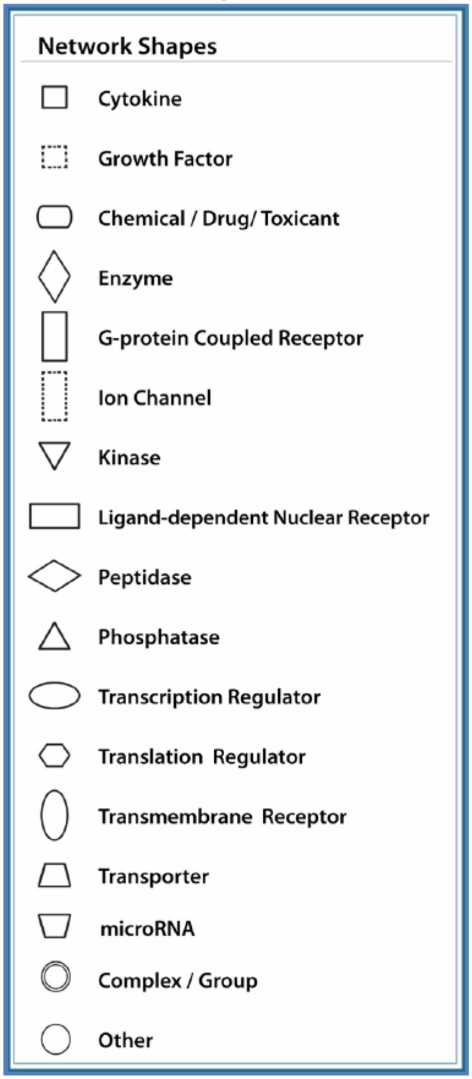

*For dataset files that contain only identifiers (i.e. no expression values), the color gray identifies the Focus Genes from that dataset.

Red	User input molecule that is upregulated (ie. has a positive (+) expression value) and whose expression value meets the user defined cutoff.
Green	User input molecule that is downregulated (ie. has a negative (-) expression value) and whose expression value meets the user defined cutoff.
Gray	User input molecule. Neither up nor down-regulated or does not meet the user-defined cutoff.
White	Molecule that is not user specified, but incorporated into the network through relationships with other molecules.
[Blue]	For canonical pathways, molecules that are members of the network being examined are outlined in blue.
