# Supplementary figures and images for: Influenza Transmission in the Mother-Infant Dyad Leads to Severe Disease, Mammary Gland Infection, and Pathogenesis by Regulating Host Responses
Source: PLoS Pathog. 2015 Oct 8;11(10):e1005173. doi: 10.1371/journal.ppat.1005173 (PMC4598190; doi:10.1371/journal.ppat.1005173)

# Milk Production Associated Genes

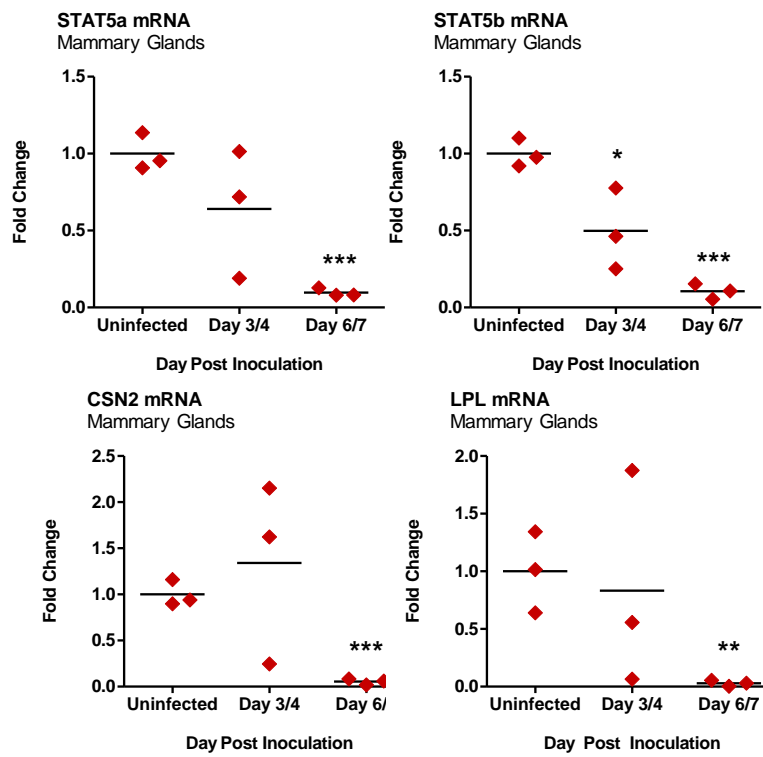

# Cancer Associated Genes

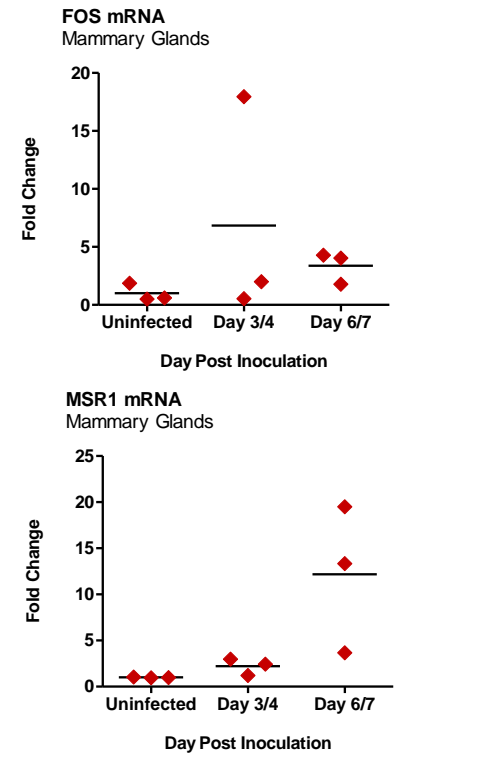

# Immune Response Genes

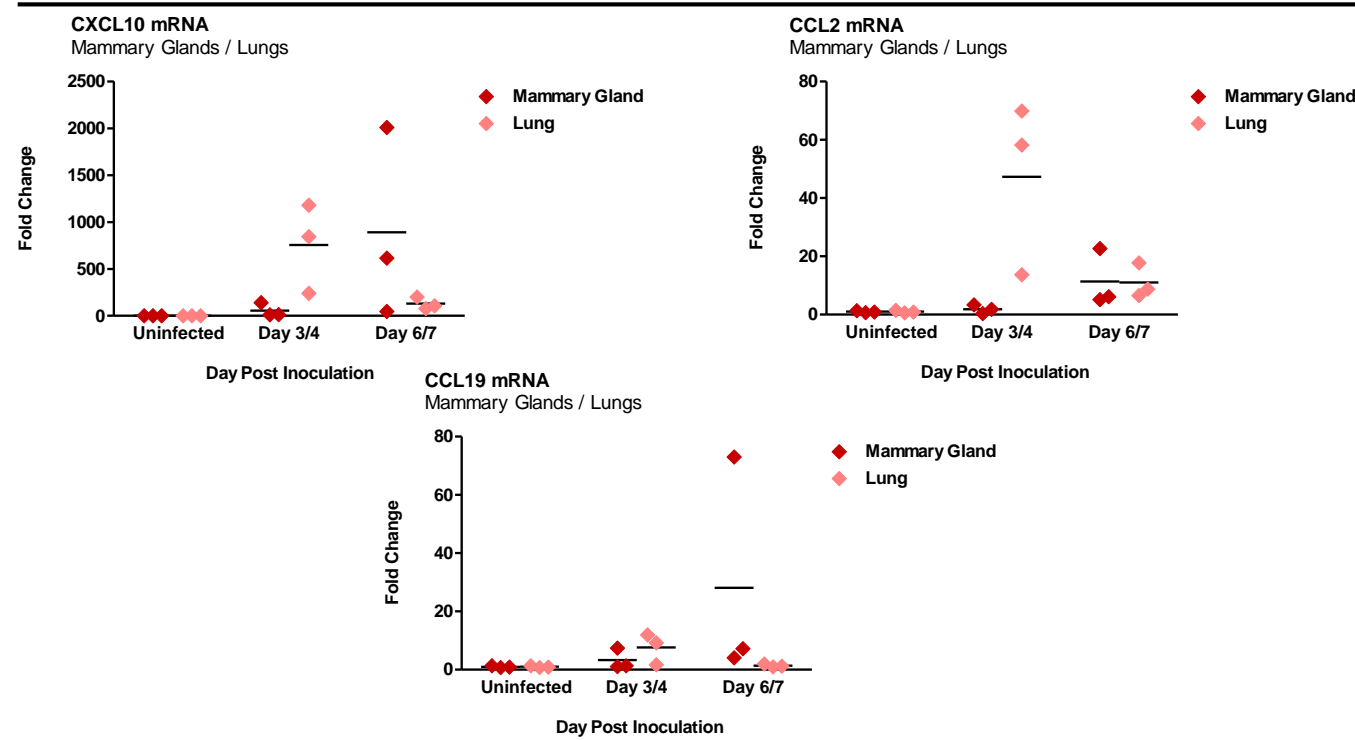

Supplement: S3 Fig — Real-Time PCR was conducted on RNA extracted from 2009 H1N1 ferret mother mammary gland for genes representing Milk Production, Cancer-Associated, and Immune Response pathways. Mammary glands were collected from nursing mothers on Day 3/4 or Day 6/7 post-infant infection and RNA was extracted. (PDF) [file ppat.1005173.s008.pdf]

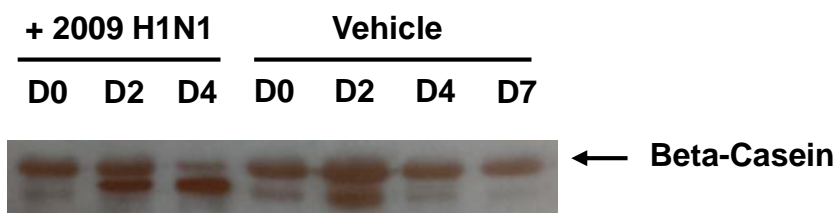

Supplement: S5 Fig — Lactating ferret mothers were inoculated intramammary gland with Cal/07 at 105 EID50. Expressed milk was electrophoresed by SDS-PAGE and analyzed by western blot with a beta-casein antibody. (PDF) [file ppat.1005173.s010.pdf]
