# Supplementary material for: Influenza Transmission in the Mother-Infant Dyad Leads to Severe Disease, Mammary Gland Infection, and Pathogenesis by Regulating Host Responses
Source: PLoS Pathog. 2015 Oct 8;11(10):e1005173. doi: 10.1371/journal.ppat.1005173 (PMC4598190; doi:10.1371/journal.ppat.1005173)
Supplement: S4 Fig — Global clustering analysis of all significantly differentially regulated genes (p-value<0.05, fold change ≥1.5 fold) at Day 3/4 and/or Day 7 in bystander mammary glands; total number of genes: 6821. Hallmark functional groups of each cluster are indicated (Ai). Gene enrichment score profiles for all KEGG-defined signaling pathways which had exhibited significant enrichment among either upregulated or downregulated gene subsets at Days 3/4 or Day 7 (see S4 Table for more detail) (Aii). Clustergrams of significantly differentially regulated genes associated with immune processes (Aiii). Comparative transcriptomic analysis of H1N1+ and bystander mammary glands. The number of significantly differentially regulated genes in H1N1+ glands only, bystander glands only, or both are indicated by Venn diagram and the most prominent functional groups for each gene subset are described (see S5 Table for more detail) (B). Upregulated genes are labeled red and downregulated genes are labeled blue throughout. Samples were collected and analyzed from six independent litter experiments (n = 3/time-point). (PDF) [file ppat.1005173.s009.pdf]

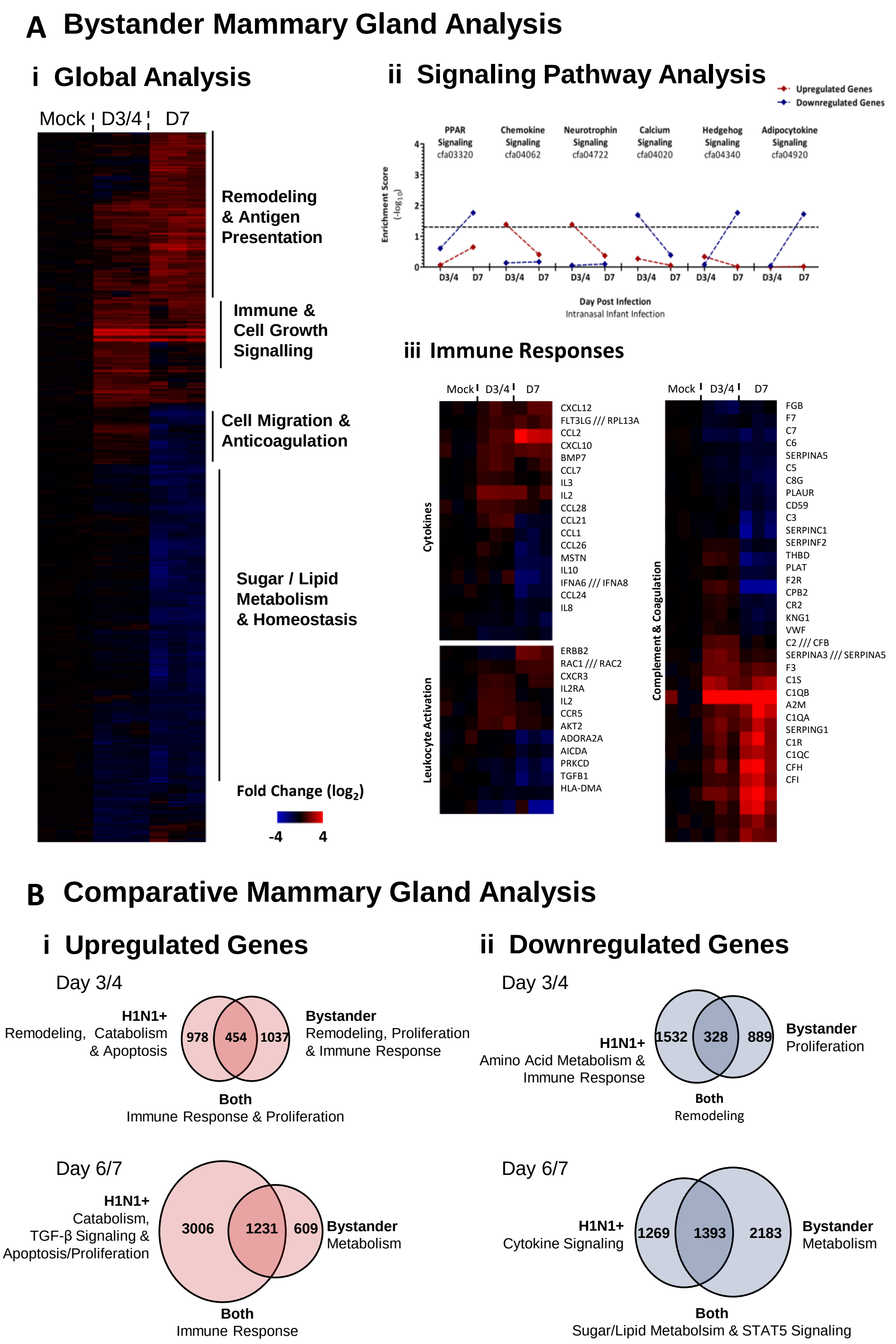
A Bystander Mammary Gland Analysis
i Global Analysis
Mock
D3/4
D7
Remodeling & Antigen Presentation
Immune & Cell Growth Signalling
Cell Migration & Anticoagulation
Sugar / Lipid Metabolism & Homeostasis
Fold Change (log2)
-4
4
ii Signaling Pathway Analysis
Upregulated Genes
Downregulated Genes
PPAR Signaling cfa03320
Chemokine Signaling cfa04062
Neurotrophin Signaling cfa04722
Calcium Signaling cfa04020
Hedgehog Signaling cfa04340
Adipocytokine Signaling cfa04920
Enrichment Score (-log10)
D3/4
D7
Day Post Infection
Intranasal Infant Infection
iii Immune Responses
Cytokines
CXCL12
FLT3LG /// RPL13A
CCL2
CXCL10
BMP7
CCL7
IL3
IL2
CCL28
CCL21
CCL1
CCL26
MSTN
IL10
IFNA6 /// IFNA8
CCL24
IL8
Leukocyte Activation
ERBB2
RAC1 /// RAC2
CXCR3
IL2RA
IL2
CCR5
AKT2
ADORA2A
AICDA
PRKCD
TGFB1
HLA-DMA
Complement & Coagulation
FGB
F7
C7
C6
SERPINA5
C5
C8G
PLAUR
CD59
C3
SERPINC1
SERPINF2
THBD
PLAT
F2R
CPB2
CR2
KNG1
VWF
C2 /// CFB
SERPINA3 /// SERPINA5
F3
C1S
C1QB
A2M
C1QA
SERPING1
C1R
C1QC
CFH
CFI
B Comparative Mammary Gland Analysis
i Upregulated Genes
Day 3/4
H1N1+
Remodeling, Catabolism & Apoptosis
978
454
1037
Bystander
Remodeling, Proliferation & Immune Response
Both
Immune Response & Proliferation
Day 6/7
H1N1+
Catabolism, TGF-β Signaling & Apoptosis/Proliferation
3006
1231
609
Bystander
Metabolism
Both
Immune Response
ii Downregulated Genes
Day 3/4
H1N1+
Amino Acid Metabolism & Immune Response
1532
328
889
Bystander
Proliferation
Both
Remodeling
Day 6/7
H1N1+
Cytokine Signaling
1269
1393
2183
Bystander
Metabolism
Both
Sugar/Lipid Metabolsim & STAT5 Signaling
